# Heading Down the Wrong Pathway: on the Influence of Correlation within Gene Sets

**DOI:** 10.1186/1471-2164-11-574

**Published:** 2010-10-18

**Authors:** Daniel M Gatti, William T Barry, Andrew B Nobel, Ivan Rusyn, Fred A Wright

**Affiliations:** 1 Department of Environmental Sciences & Engineering, Gillings School of Global Public Health, University of North Carolina at Chapel Hill, Chapel Hill, NC, USA; 2 Department of Biostatistics and Bioinformatics, Duke University School of Medicine, Durham, NC, USA; 3 Department of Statistics and Operations Research, University of North Carolina at Chapel Hill, Chapel Hill, NC, USA; 4 Department of Biostatistics, University of North Carolina at Chapel Hill, Chapel Hill, NC, USA; 5 Centers for Environmental Bioinformatics and Computational Toxicology, University of North Carolina at Chapel Hill, Chapel Hill, NC, USA

## Abstract

**Background:**

Analysis of microarray experiments often involves testing for the overrepresentation of pre-defined sets of genes among lists of genes deemed individually significant. Most popular gene set testing methods assume the independence of genes within each set, an assumption that is seriously violated, as extensive correlation between genes is a well-documented phenomenon.

**Results:**

We conducted a meta-analysis of over 200 datasets from the Gene Expression Omnibus in order to demonstrate the practical impact of strong gene correlation patterns that are highly consistent across experiments. We show that a common independence assumption-based gene set testing procedure produces very high false positive rates when applied to data sets for which treatment groups have been randomized, and that gene sets with high internal correlation are more likely to be declared significant. A reanalysis of the same datasets using an array resampling approach properly controls false positive rates, leading to more parsimonious and high-confidence gene set findings, which should facilitate pathway-based interpretation of the microarray data.

**Conclusions:**

These findings call into question many of the gene set testing results in the literature and argue strongly for the adoption of resampling based gene set testing criteria in the peer reviewed biomedical literature.

## Background

Methods for statistical analysis of gene expression microarrays are maturing rapidly, and there are a variety of approaches to normalization, detection of differential expression, clustering, and class prediction [1]. In many experiments, a statistical test is performed to identify genes significantly associated with experimental condition, clinical response, or other sample attributes. The resulting list of significant genes may be so large that it defies easy interpretation, and it is natural to seek a concise, biological summary of results. One such approach is gene set testing (sometimes called "pathway analysis"), which detects over-representation of gene sets among the list of significant genes. Gene sets may be curated [2], or derived from databases such as Gene Ontology (GO) [3] or Kyoto Encyclopedia of Gene and Genomes (KEGG) [4].

**Figure 1:**
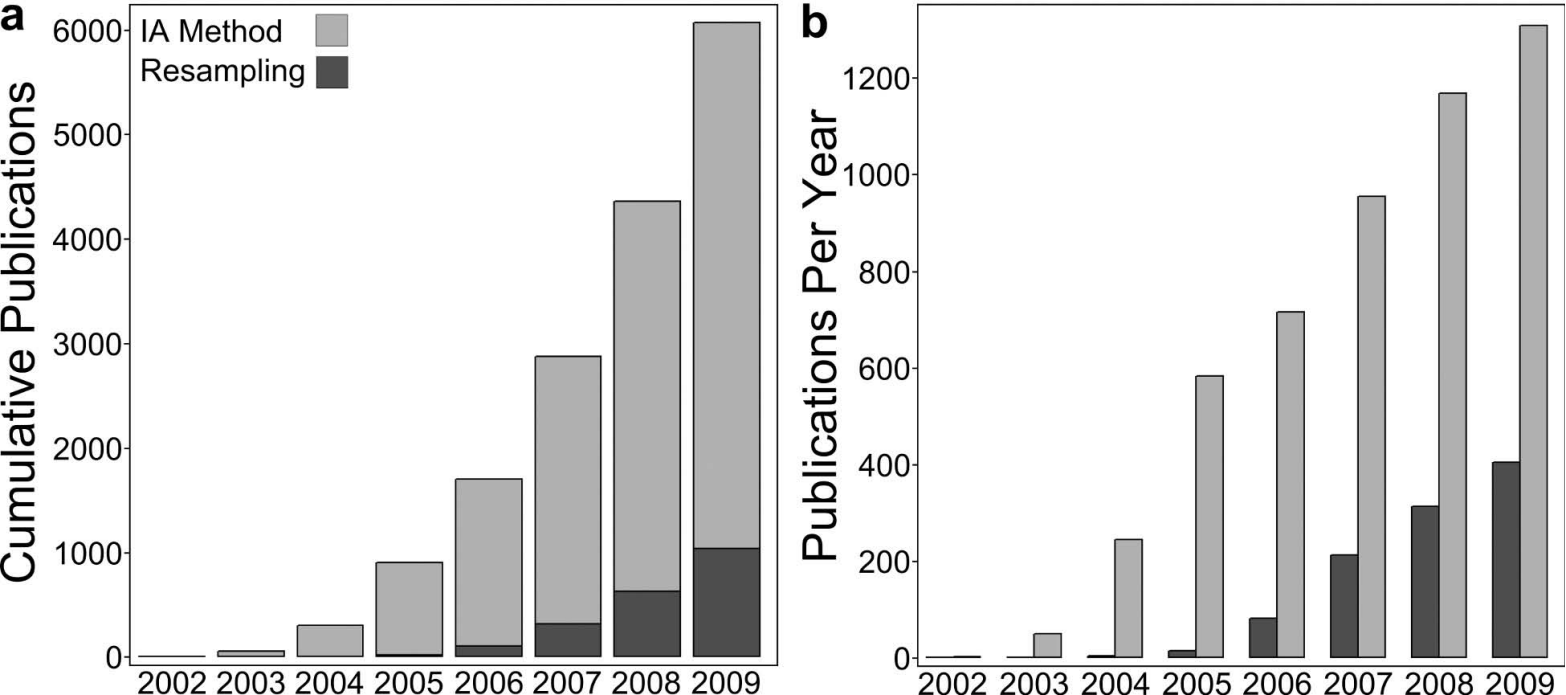
**Publications that assume independence between genes (light grey) greatly outnumber publications that use array resampling methods (dark grey)**. Panel (a) shows the cumulative number of publications and panel (b) shows the number of publications using each method per year. Year of publication is displayed on the horizontal axis.

The simplest approach to gene set testing relies on 2 × 2 tables of gene set membership (in gene set or not) vs. significance (significant or not). Gene set testing is often performed using a χ^2 ^or Fisher's Exact test [5], which rely critically upon the assumption that individual genes, and their associated test statistics, are independent, hereafter referred to as the *independence assumption *[6]. Many software tools use independence assumption methods (reviewed in [7]) and these tools are employed by the majority of publications using gene set testing (Figure 1a). However, independence of genes is a false assumption for expression microarrays: indeed, correlation forms the basis for informative techniques such as hierarchical clustering [8,9]. Several authors have noted, via simulation or analysis of a few experimental data sets, that correlation adversely affects the selection of significant gene sets [6,10-13]. Investigators who have recognized this problem employed resampling-based approaches that maintain the correlation structure of the expression data and produce an empirical estimate of gene set significance [14,15]. Resampling approaches have also been integrated into many software tools to address this problem [2,10,16-19]. Nevertheless, the use of independence assumption-based methods continues to increase (Figure 1b).

As the independence assumption is clearly violated for gene expression microarrays [11,20,21], how can we explain the persistent use of independence methods? Independence assumption methods may appeal to the belief that the occurrence of multiple members of a gene set on a list of significant ones "cannot be due to chance." Indeed, in a post-hoc interpretation, the fact that the genes within a set are correlated is often, incorrectly, viewed as *reinforcing *the biological plausibility [5,22], despite evidence presented here and elsewhere that the presence of correlation increases false-positive rates. It is conceivable to argue that correlation within a gene set itself provides additional information, if such correlation varied substantially across different experiments. However, as we show here, correlation within a gene set is largely a persistent property that is preserved across a wide variety of sample sources and experimental conditions.

Here, we clearly demonstrate that the correlation between genes inflates the false positive rate, that the magnitude of this inflation is quite high, and that a simple resampling method for gene set testing produces the correct false positive rate. We use a large number (over 200) of real experimental datasets, not simulated data, to show that 1) inter-gene correlation is a pervasive feature of gene sets, regardless of experimental condition, 2) inter-gene correlation inflates the apparent significance of gene set statistics, leading to an increase in false positives, and 3) array resampling approaches can correctly address the problem of inter-gene correlation. As there are several existing tools that use resampling approaches to determine functional enrichment among significant gene lists, we argue that the naïve approaches should no longer be used and should be replaced by tools employing a resampling approach.

## Results

**Figure 2:**
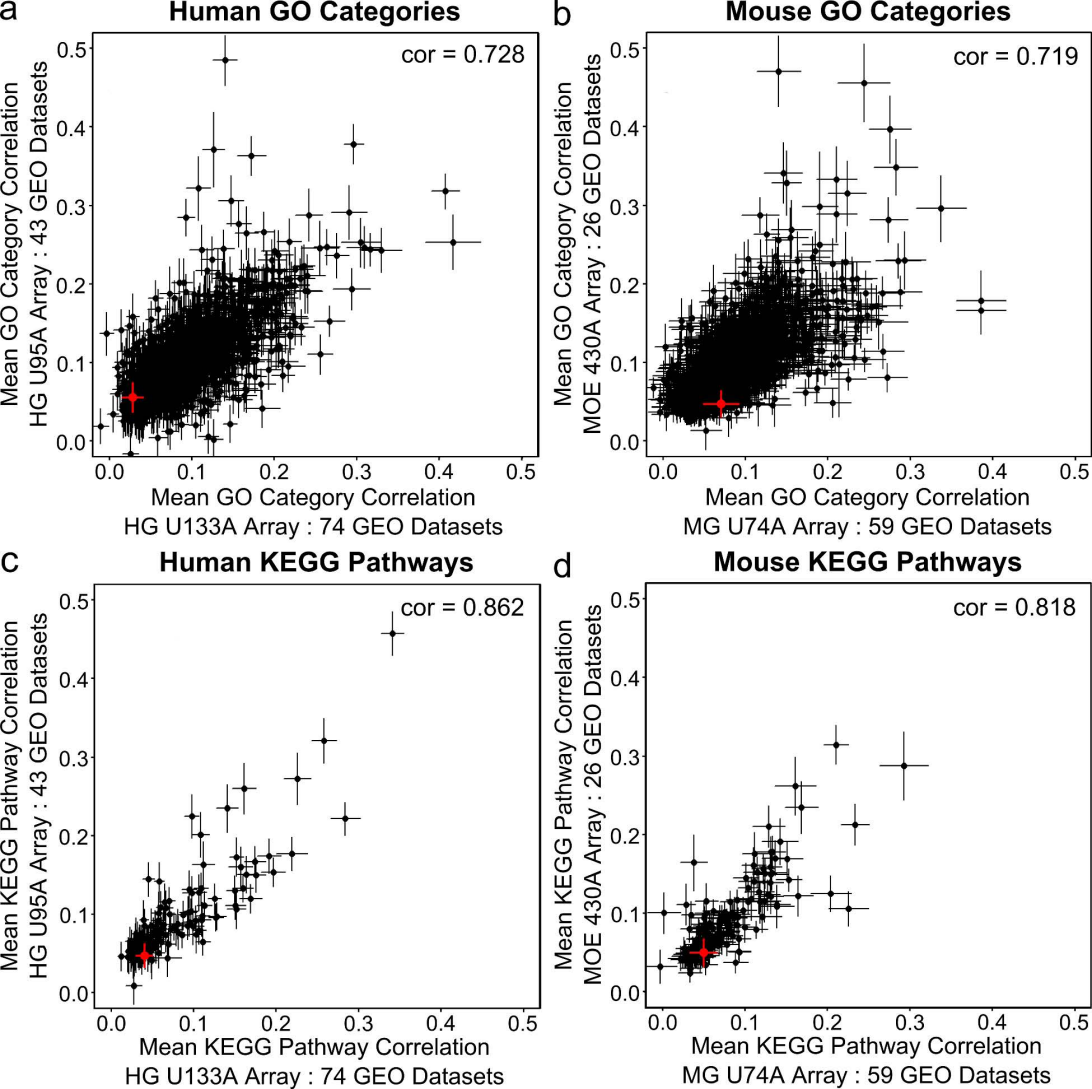
**The inter-gene correlation within GO categories is consistent across experiments and platforms**. Mean correlation among genes in GO categories (a,b) and KEGG pathways (c,d) on two human (a,c) and two mouse (b,d) microarray platforms. The correlation of all transcripts with all transcripts on each platform is shown in red. Spearman correlations of the correlations are in upper right. Crosses represent +/- 1 standard error on each axis.

We investigated the degree to which correlation within gene sets is preserved across multiple experiments by analyzing Gene Expression Omnibus [23] data from two human (HG-U133A, HG-U95A), and two mouse (MG-U74A, MOE430A) arrays. Datasets with 20 or more samples were used, resulting in 202 datasets and 8,656 arrays. For each dataset we calculated the mean correlation for genes within each GO category or KEGG pathway. The results give strong evidence of the reproducibility of internal correlations (low standard errors in Figure 2), with strong agreement of gene set correlations between platforms (Spearman cor.: 0.719 - 0.862) (Figure 2). Correlation within gene sets is thus closely associated with gene set membership, regardless of experimental condition [9]. The samples represent a wide variety of experiments, including *in vitro *cell lines, sex comparisons, multiple cancer sub-types, tissues, and diseases. Clearly, in contrast to previous assertions [5,22], mere correlation of genes within gene sets and pathways is commonplace, and does not necessarily reflect unique features of the experiment at hand. As Figure 2 shows, correlation within gene sets covers a wide range, and is generally higher than correlation of randomly selected genes. The correlations within gene sets are much higher than the correlation of all transcripts on each platform with each other (Figure 2, red). Of course, genes from different sets can be correlated (even highly so), but we confine our focus here to pre-defined gene sets.

One simple way to describe the effect of correlation is through a standardized enrichment test statistic. Let *z *be the signed square root of the independence assumption χ^2 ^statistic (see Methods). Under the null hypothesis (no enrichment), *z *should have approximately mean 0 and variance 1. However, the true variance can be much greater, with an increase proportional to (*m *- 1)*ρ *(see Methods), where *m *is the number of genes in the set, and *ρ *is the average correlation among genes in the set (excluding self-correlations). Independence assumption methods assume *ρ *= 0, implying that the variance equals 1. However, if the average internal correlation of a gene set is positive, then the variance of *z *can be greatly inflated, even if this correlation is modest, depending on the gene set size *m*.

To empirically demonstrate that variance inflation results in the false significance of gene sets, we randomly permuted the sample labels associated with each of the 202 Gene Expression Omnibus data sets 10,000 times, and then performed a gene set analysis on each permutation using a common independence assumption method. For each permutation a list of "significant" genes with *p *≤ 0.05 (using a t-test or F-test, as appropriate) was created in order to have a meaningfully-sized gene list, and the standard χ^2 ^statistic was used to assess the significance of each gene set. A gene set was called significant if it had a Bonferroni-corrected (for the number of sets) χ^2 ^*p*-value ≤ 0.05. Note that this is a highly conservative threshold, intended to guarantee that no more than 5% of the permutations result in one or more gene sets (falsely) called significant. The gene set null hypothesis is that each gene set has the same pattern and proportion of differentially expressed genes as any other set [24]. Permutation of the sample labels induces the null hypothesis for each gene, and thus no gene sets should exhibit enrichment for differential expression. Hence, any gene set declared significant is a false positive.

**Figure 3:**
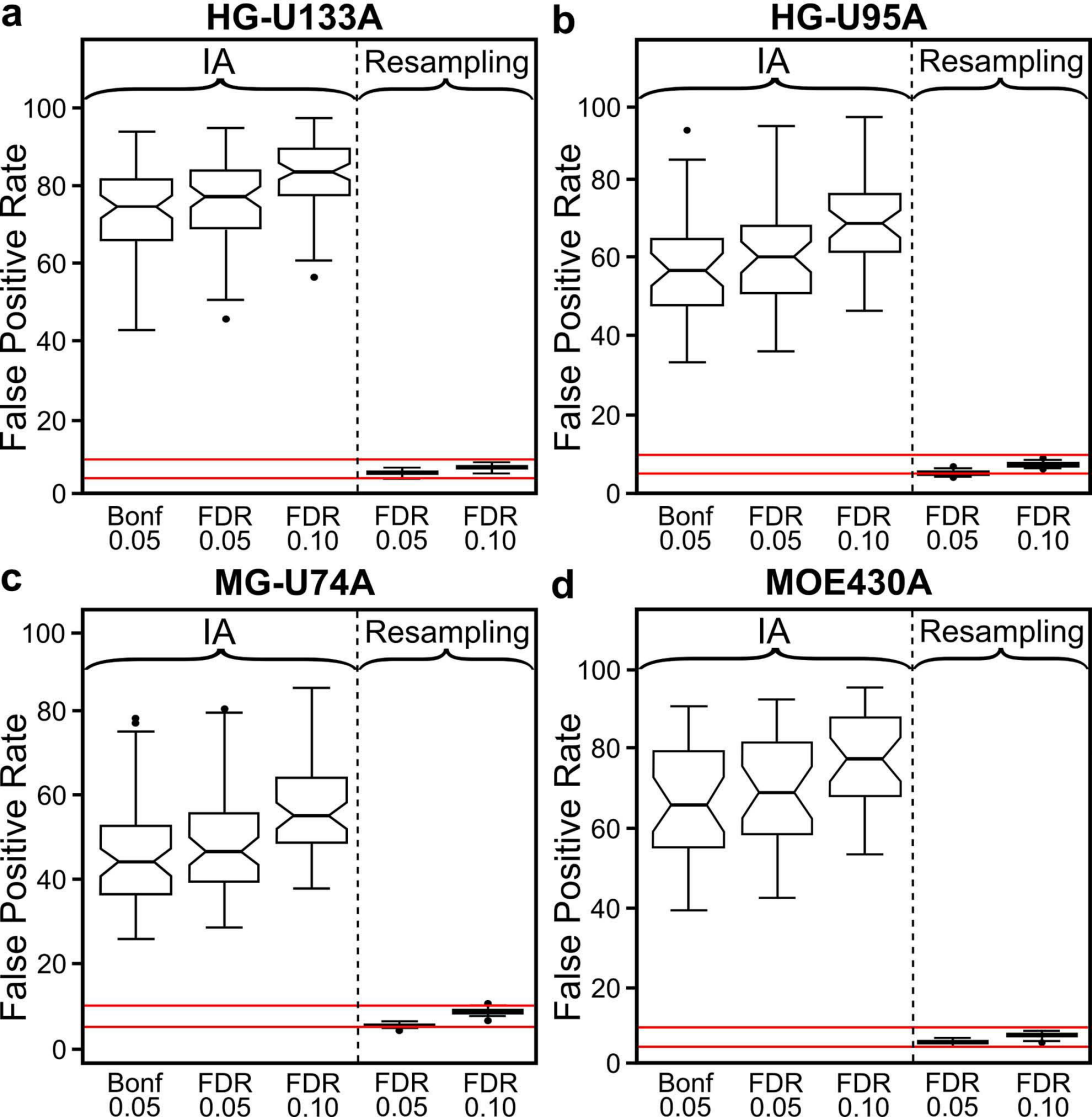
**False positive rates are greatly increased using independence assumption methods, GO Biological Process categories**. The proportion of permutations in which at least one GO Biological Process category is called significant using an independence assumption method with a Bonferroni correction (α = 0.05), the Benjamini & Hochberg FDR (α = 0.05, 0.10), and the resampling approach described in this manuscript. Red lines = 5% & 10%.

To determine the overall experiment-wise increase in false positives, we counted the number of permutations in which at least one gene set was declared significant, using the Bonferroni-corrected 0.05 threshold. If the independence assumptions were true, no more than 5% of the permutations would give rise to significant gene sets. However, the observed proportion is much higher (Figure 3; Additional file 1, Figure S1). False positive rates often exceed 50%, and in some cases are above 80%, and are even higher when the False Discovery Rate (FDR) is used as a multiple comparison correction. Clearly such high false positive rates have serious implications for the conclusions drawn by any study which uses independence assumption methods to provide biological interpretation of microarray data.

**Figure 4:**
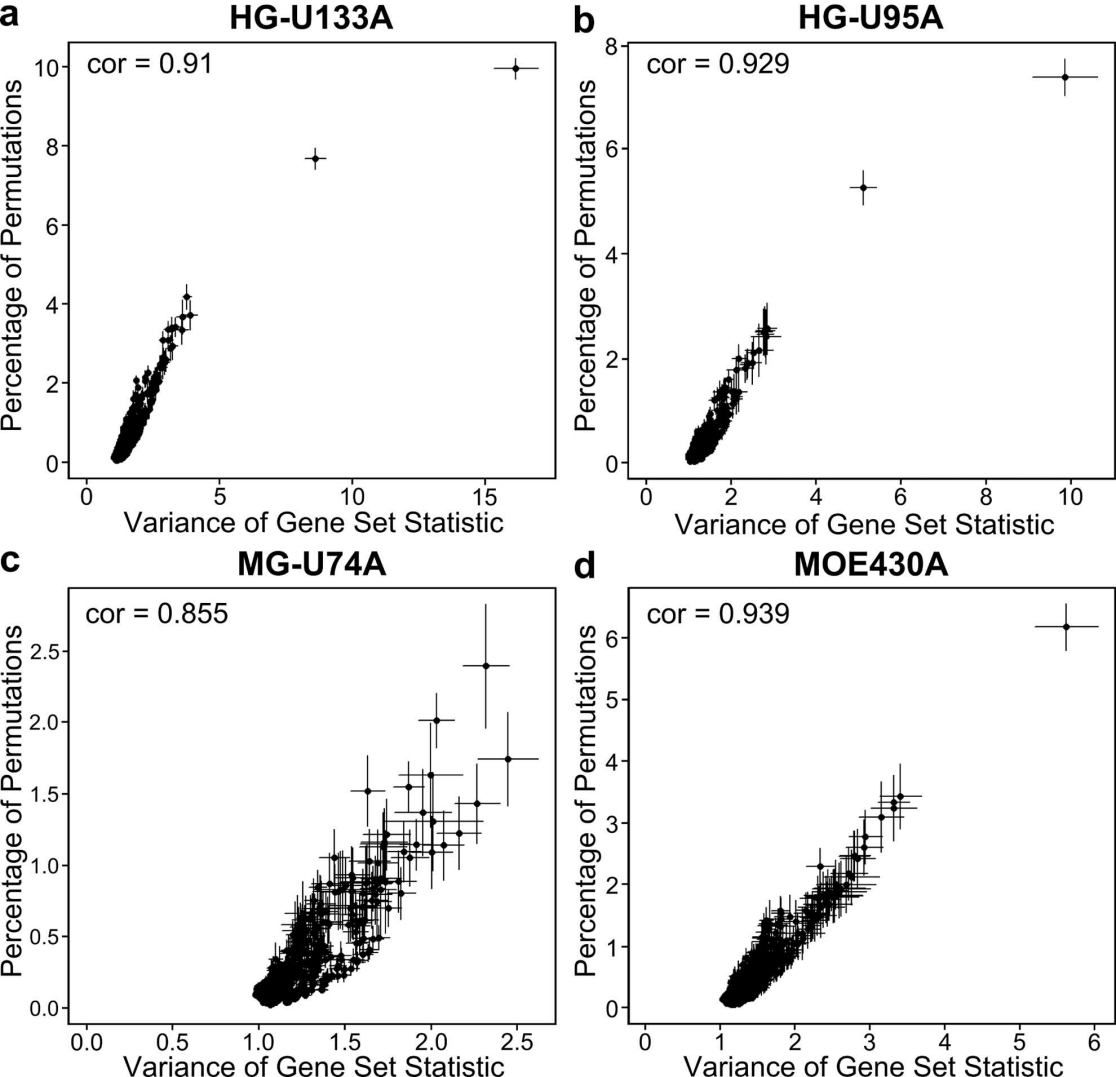
**Variance inflation due to correlation of gene expression increases the false positive rate, even when using a Bonferroni correction**. The percentage of permutations in which at least one GO Biological Process category was called significant is shown versus the variance of the gene set statistic, for two human (a,b) and two mouse (c,d) arrays. Spearman correlations are in the upper left of each panel.

In addition, as predicted, we found that variance inflation is directly related to the false-positive rate for a gene set. For each GO Biological Process (Figure 4) or KEGG pathway (Additional file 1, Figure S2), we show that the empirical variances of the standardized enrichment statistic (which often greatly exceed 1) are highly correlated with the per-category false positive rates. Specifically, for each gene set, we calculated the variance of the square root of the χ^2 ^statistic across all permutations, and plotted it against the number of permutations in which that gene set was called "significant" using a Bonferroni correction.

Fortunately, the shortcomings of independence assumption methods can be addressed through the use of resampling-based methods. These methods use the resampling data to construct an empirical null distribution for the gene set test statistics, taking into account the correlation structure between genes and providing a more accurate assessment of statistical significance. Several existing tools use resampling based approaches [2,16-19], either permuting sample labels relative to gene expression, or performing bootstrap resampling [24]. We empirically investigated the performance of the permutation approach, which we will refer to as "resampling" to avoid confusion with the permutations described above. The sample labels for each data set were resampled without replacement 10,000 times, and the resulting enrichment statistic distribution was used to obtain an empirical p-value for each gene set. For example, if the observed enrichment statistic for a gene set was greater than or equal to 99% of the resampled enrichment statistics, a p-value of 0.01 was assigned to that gene set. As the resampling units are entire arrays, correlation between genes is maintained under resampling. Therefore the effects of correlation within gene sets apply to both the resampled and observed data, properly controlling the false positive rate. To demonstrate that the resampling approach correctly controls the false positive rate, we applied the resampling procedure to all permutations of all 202 datasets, using the same χ^2 ^statistic as above. Using the empirical distribution of test statistics under resampling to generate the gene set p-value, proper control of the false positive rate was obtained (Figure 3; Additional file 1, Figure S1, right-most boxplots).

**Figure 5:**
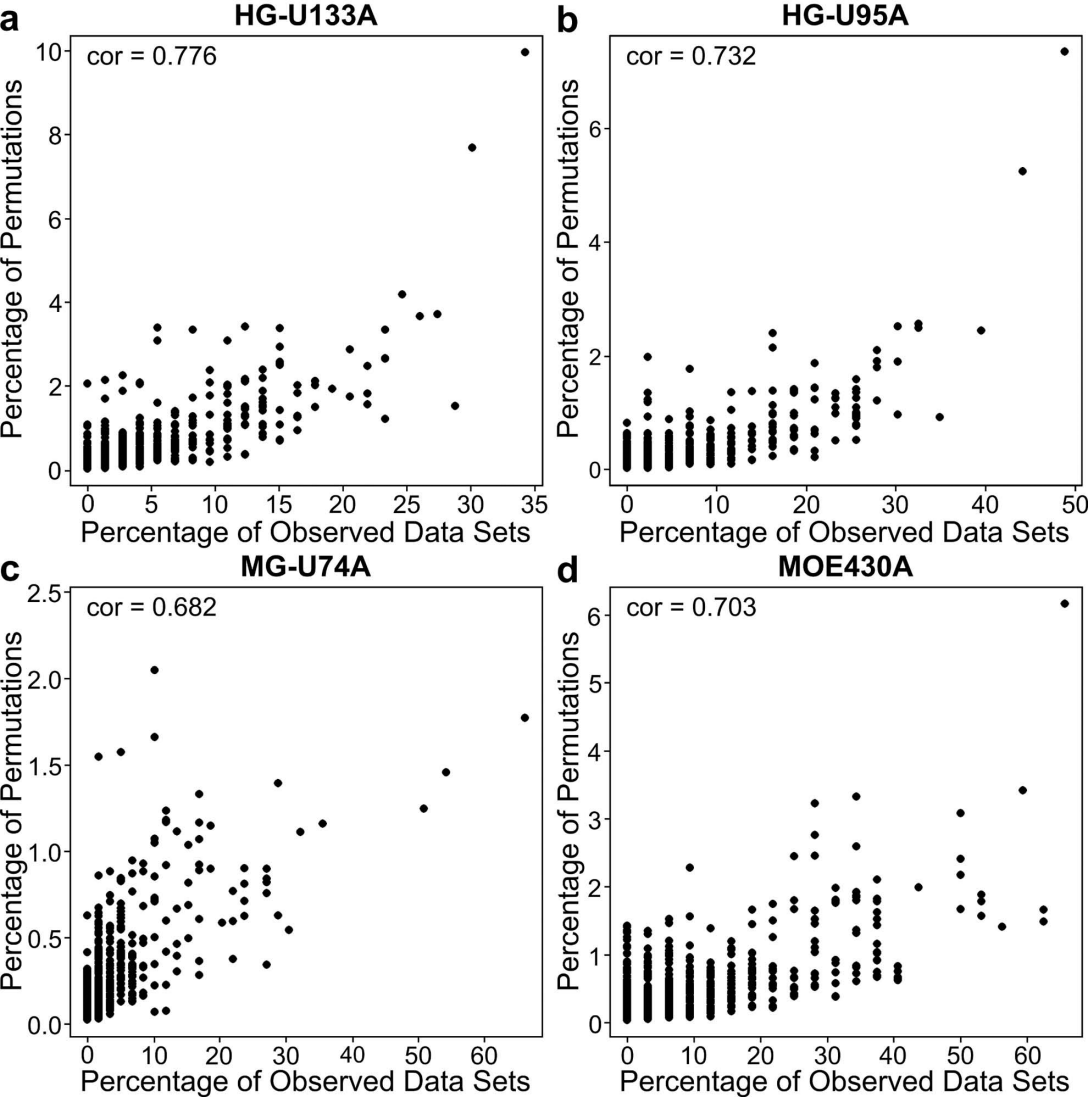
**GO Biological Process categories that are called significant by chance under permutation are likely to be called significant in the observed data**. The proportion of times that a category is declared significant under permutation is plotted versus the proportion of times it is called significant in the observed data. Spearman correlations in upper left corner.

While the analyses above clearly show that gene correlation increases the false positive rate for independence assumption methods, it may be tempting to argue that correlation does not affect false positive rates in real datasets, which presumably include true enrichment. To investigate this, we computed the proportion of observed datasets in which each gene set was declared significant by the independence assumption method, and compared these values to the previously generated proportions observed under random permutation. The results for GO Biological Process categories (Figure 5) and KEGG pathways (Additional file 1, Figure S3) demonstrate a strong relationship. In other words, categories declared statistically significant by independence assumption methods in actual datasets are often declared significant when treatment assignments are made purely at random. This strongly calls into question the biological conclusions drawn from such analyses, as we have demonstrated that such correlation is a predominant source of misinterpretation, rather than a biological confirmation. Indeed, some gene sets have such high false positive rates (Figures 3 and 5) under independence assumption that one or more sets may be found significant in multiple studies, further enhancing apparent biological confirmation.

## Discussion

The most straightforward strategy for gene expression analysis is to focus on individual genes for which expression differs among samples of interest. Such approaches often consider the genes selected based on significance thresholds, with the goal of predicting or finding associations with disease prognosis [25], adverse response to a chemical [26], disease pathogenesis [27], or identification of key genes in a pathway that may serve as biomarkers [28]. Although useful, gene-by-gene analyses may not account for the complex underlying biology where the transcriptional programs are distributed across an entire network of genes, and may involve only subtle changes among individual genes. Even for gene-by-gene analysis, the presence of strong correlations is increasingly recognized as a potentially complicating feature. For example, surrogate variable analysis [29] attempts to reconstruct latent variables which explain variation in gene expression, which rely on gene-gene correlation in an essential manner.

Gene set testing, in which enrichment of significant differentially expressed genes is sought among gene sets, is by now a standard method to provide biological interpretation for gene expression data. Groups of genes with a common biological function, cellular localization, regulation, or chromosomal location may hold additional clues regarding underlying biology, or potentially improve prediction or classification. While many easy-to-use tools have been developed to facilitate pathway- or gene set-based analysis, inter-gene correlation within gene sets violates the independence assumption that underlies many gene set analysis methods. Indeed, our meta-analysis demonstrates that the correlation patterns persist across a wide variety of mouse and human experiments. Thus, we argue that correlation patterns should largely be viewed in terms of their effects on false positives, as we find no evidence that correlation within the gene set confers additional plausibility to a gene set finding.

Though it may be viewed as a technical matter, violation of the independence assumption is not a mere statistical detail. It is a tangible phenomenon that increases the chance of falsely declaring a gene set significant. The false positive rates established by our study are very high - sometimes an order of magnitude beyond the intended false positive rate. Array resampling methods correctly handle inter-set correlation and are not subject to these high false positive rates. Barry et al. [24] describe investigations of power and provides context on the meaning of type I error control for resampling-based methods. The null distribution induced by permutation here is a special case of the expanded definition in [24], because the null hypothesis holds for each gene. Bootstrap resampling methods were shown in [24] to have more power than array permutation methods in simulated and real data sets, and expansions beyond the existing resampling approaches are worthy of investigation. There are a wide number of available packages that implement these methods, such as GSEA [2], Catmap [16], SAFE [17], ErmineJ [10], and SAM-GS [18].

Several methods have been proposed which use multivariate modeling of array data to perform gene set testing [19,30,31]. Although comparisons using such approaches were not a main focus, we briefly tested whether these methods correctly control the false positive rate, by permuting the sample labels for one data set (GDS266) 1,000 times and counting the number of permutations in which at least one GO BP category was called significant using a 5% FDR. The globaltest procedure [19] uses a score-based test statistic to test for correlation of an entire set of genes with clinical outcome, and can be used for gene set testing. The parametric version of globaltest incorporates the observed correlation structure, and is compared to a χ^2 ^distribution. However, we found that 94% of permutations called at least one GO BP category significant, with a median of 10 categories called significant in each permutation, suggesting difficulties in proper control of the false positive rate. It is worth noting that use of empirical covariance estimates is extremely challenging, especially when gene sets are larger than the sample size. GlobalANCOVA [30] fits a linear model between each gene and the experimental covariates, and uses permutation testing to obtain empirical p-values. We found that GlobalANCOVA found no significant GO BP categories under permutation, suggesting that GlobalANCOVA properly controls the false positive rate, and is perhaps conservative. Several related methods have been proposed [31,32] that test for differential expression or predictive ability of sets of genes. Many of these approaches attempt to incorporate gene-gene correlation structure parametrically, and are thus computationally appealing. However, until more comprehensive investigations have been performed for the multivariate procedures, it seems prudent to examine the false positive rate in 1,000 permutations of their data before accepting the results at face value. Other investigators have explored more complex procedures involving stochastic dependence [33], while deeper explorations of random-set methods [34] may be valuable, but still essentially rely on independence assumptions. Several other methods are reviewed and compared in [35-37]. In summary, despite the availability of software and a wide array of methods that address inter-gene correlation, the literature on microarray analysis is rife with studies that use independence assumption methods.

As the use of independence assumption methods in gene array studies is widespread, we conclude that published false gene set findings may be common. The precise impact of the overly optimistic statistical support for many gene set findings is difficult to assess, because we do not know the underlying truth; however, a basic requirement of a valid statistical test is that it controls false positives under the null hypothesis, and we have clearly demonstrated here that methods that rely on independence assumptions are therefore invalid in this sense.

It should be acknowledged that very small microarray studies may have too few samples to support permutation. For such studies, a strong statistical result from an independence assumption-based method should be supported by corroborating biological evidence, and even then be interpreted with caution.

## Conclusions

Here, we have demonstrated, using over 200 real experimental data sets that independence assumption methods for gene set enrichment suffer from such a high false positive rate that they should not generally be used. Array resampling methods, which correctly control the false positive rate, should be used by investigators, incorporated into existing routines and workflows, and insisted upon by reviewers of scientific manuscripts. We strongly encourage the use of tools such as GSEA [2], SAFE [17], Catmap [16], ErmineJ [10], SAM-GS [18] or others which employ resampling-based methods to determine gene set significance.

## Methods

### Compilation and Categorization of Pathway Tools

Using the list of pathway tools compiled by [7], we downloaded all citations for each tool from the ISI Web of Knowledge (http://apps.isiknowledge.com/, accessed March 31, 2010). The manuscripts (for the period from 2002 to 2009) were divided into those using resampling- or independence assumption-based methods as detailed in [7]. Reviews and manuscripts that did not explicitly state the pathway selection method were not counted.

### Gene Expression Omnibus Datasets

Using the GEOmetadb [38] and GEOquery [39] packages from Bioconductor, Gene Expression Omnibus was queried in April 2009 for all datasets run on the Affymetrix (Santa Clara, CA) HG-U133A, HG-U95A, MG-U74A and MOE430A that contained 20 or more samples. There were 74, 43, 59 and 26 datasets for each array, respectively. The data was used as normalized by the submitters. The datasets are listed in Additional file 2, Tables S1 through S4.

### Correlation Analysis and Variance Inflation

For each array platform, the genes in each GO category from all ontologies and each KEGG pathway with between 5 and 5000 genes were collected. The mean Pearson correlation was taken as the mean pairwise correlation between all genes in the gene set, excluding the unit correlation of each gene with itself. For each gene set, the mean and standard error of the correlations in all datasets for a single platform was calculated. The variance inflation factor, used here only to provide motivation for our empirical results, is derived as follows. We construct the 2 × 2 table of gene significance (using nominal *p*-value threshold *α*) and membership in a gene set of size *m*. A valid statistic, and approximately equivalent to the standard χ^2 ^statistic, can be constructed from the single table entry for "significant and in the gene set," assuming that no substantial enrichment is expected in the remaining genes. Using *i *to index the genes in the set, we have observed entry $O=\sum_{i=1}^{m}I[p_{i}\leq \alpha ]$, which has variance

$$\sum_{i=1}^{m}\alpha (1-\alpha )+\sum_{i\neq j}\mathrm{cov}(I[p_{i}\leq \alpha ,p_{j}\leq \alpha ])=m\alpha (1-\alpha )+(m^{2}-m)\;\bar{\text{corr}}(I[p_{i}\leq \alpha ],I[p_{j}\leq \alpha ])\;\alpha (1-\alpha ),$$

where $\bar{\text{corr}}(I[p_{i}\leq \alpha ],I[p_{j}\leq \alpha ])$ is the average correlation of the significance indicators for different genes *i *and *j *in the gene set. Dividing through by the independence assumption variance (${\mathrm{var}}_{\text{IA}}=\sum_{i=1}^{m}\alpha (1-\alpha )$), we obtain $\mathrm{var}(O)/{\mathrm{var}}_{\text{IA}}(O)=1+(m-1)\;\bar{\text{corr}}(I[p_{i}\leq \alpha ],I[p_{j}\leq \alpha ])$. The average correlation of significance indicators has a nonlinear relationship with the original within-set gene-gene mean correlation *ρ*. However, observed variance inflation values are roughly proportional to *mρ*, as illustrated in Additional file 1, Figure S4. The concept of variance inflation is described more extensively in [24].

### Independence Assumption Analysis

GO Biological Process categories and KEGG pathways with between 25 and 5000 genes were used to satisfy the assumptions of the χ^2 ^test. Based on the experimental annotation provided in Gene Expression Omnibus for each dataset, either a Student's T-test or analysis of variance (ANOVA) was carried out on each gene to select differentially expressed genes between the experimental classes provided in the annotation. Significant genes were selected using a nominal *p*-value of 0.05. A one-sided standard χ^2 ^test (i.e. to test for enrichment only) was performed on each gene set, and significant gene sets were selected at a Bonferroni corrected *p*-value of 0.05. Both GO Biological Process categories and KEGG pathways were analyzed using this approach.

### Permutation Analysis

For each dataset, either a Student's T-test or ANOVA was carried out on each gene to select differentially expressed genes between experimental classes at a nominal *p*-value of 0.05. The one-sided χ^2 ^test statistic was used for gene set testing. The sample labels on the arrays were then permuted, and the entire analysis was repeated 10,000 times, resulting in a matrix of gene set statistics with one row for each gene set and one column for each permutation.

### Resampling Analysis

The data was permuted as above and the permutation matrices containing gene set statistics were retained. Empirical p-values for each gene set were calculated as the proportion of permutations for which the χ^2 ^statistic was greater than or equal to the observed χ^2 ^statistic. A gene set was called significant if its Benjamini & Hochberg FDR adjusted *p*-value was 0.05 or 0.10. GO Biological Process categories and KEGG pathways were both analyzed using this approach.

*Statistical Analyses *were conducted using R, version 2.8.1 [40] and array annotation from Bioconductor, version 2.3 [41].

## Abbreviations

GO: Gene Ontology; KEGG: Kyoto Encyclopedia of Genes and Genomes; FDR: False Discovery Rate; ANOVA: Analysis of Variance

## Competing interests

The authors declare that they have no competing interests.

## Authors' contributions

DG, WB and FW participated in the design of the study; DG carried out the statistical analyses and drafted the manuscript; AN, FW, and IR were involved in the analysis of the data, drafting the manuscript and revising it critically for important intellectual content. All authors have given final approval of the version to be published.

## Supplementary Material

Additional file 1 **Additional file 1, Figure S1 False positive rates are greatly increased using independence assumption methods, KEGG pathways**. The proportion of permutations in which at least one KEGG pathway is called significant using an independence assumption method with a Bonferroni correction (α = 0.05), the Benjamini & Hochberg FDR (α = 0.05, 0.10), and the resampling approach described in this manuscript. **Additional file 1, Figure S2**. Variance inflation due to gene expression correlation increases the false positive rate, even when using a Bonferroni correction. The percentage of permutations in which at least one KEGG pathway was called significant is plotted versus the variance of the standardized gene set statistic (signed square root of the χ^2 ^statistic). Results are shown for two human (a,b) and two mouse (c,d) arrays. **Additional file 1, Figure S3**. KEGG pathways that are called significant by chance under permutation are likely to be called significant in the observed data. The proportion of times that a KEGG pathway is declared significant under permutation is plotted versus the proportion of times it is called significant in the observed data. **Additional file 1, Figure S4**. The variance of the gene set statistic (signed square root of χ^2 ^statistic) increases in proportion to the variance inflation factor (VIF = 1 + (*m*-1)ρ). The VIF is plotted versus the variance of the gene set statistic versus for two human (a, b) and two mouse (c, d) arrays. Spearman correlations are shown in the upper right corner. Click here for file

Additional file 2 **Additional File 2. Table S1. HG-U95A array datasets**. PubMed, GEO and other IDs and descriptions of the datasets used. **Additional File 2. Table S2**. HG-U133A array datasets. PubMed, GEO and other IDs and descriptions of the datasets used. **Additional File 2**. Table S3. mgu74a array datasets. PubMed, GEO and other IDs and descriptions of the datasets used. **Additional File 2. Table S4**. moe430a array datasets. PubMed, GEO and other IDs and descriptions of the datasets used. Click here for file
