# Supplementary material for: Heading Down the Wrong Pathway: on the Influence of Correlation within Gene Sets
Source: BMC Genomics. 2010 Oct 18;11:574. doi: 10.1186/1471-2164-11-574 (PMC3091509; doi:10.1186/1471-2164-11-574)
Supplement: Additional file 1 — , Figure S1 False positive rates are greatly increased using independence assumption methods, KEGG pathways. The proportion of permutations in which at least one KEGG pathway is called significant using an independence assumption method with a Bonferroni correction (α = 0.05), the Benjamini & Hochberg FDR (α = 0.05, 0.10), and the resampling approach described in this manuscript. Additional file 1, Figure S2. Variance inflation due to gene expression correlation increases the false positive rate, even when using a Bonferroni correction. The percentage of permutations in which at least one KEGG pathway was called significant is plotted versus the variance of the standardized gene set statistic (signed square root of the χ2 statistic). Results are shown for two human (a,b) and two mouse (c,d) arrays. Additional file 1, Figure S3. KEGG pathways that are called significant by chance under permutation are likely to be called significant in the observed data. The proportion of times that a KEGG pathway is declared significant under permutation is plotted versus the proportion of times it is called significant in the observed data. Additional file 1, Figure S4. The variance of the gene set statistic (signed square root of χ2 statistic) increases in proportion to the variance inflation factor (VIF = 1 + (m-1)ρ). The VIF is plotted versus the variance of the gene set statistic versus for two human (a, b) and two mouse (c, d) arrays. Spearman correlations are shown in the upper right corner. [file 1471-2164-11-574-S1.DOC]

**
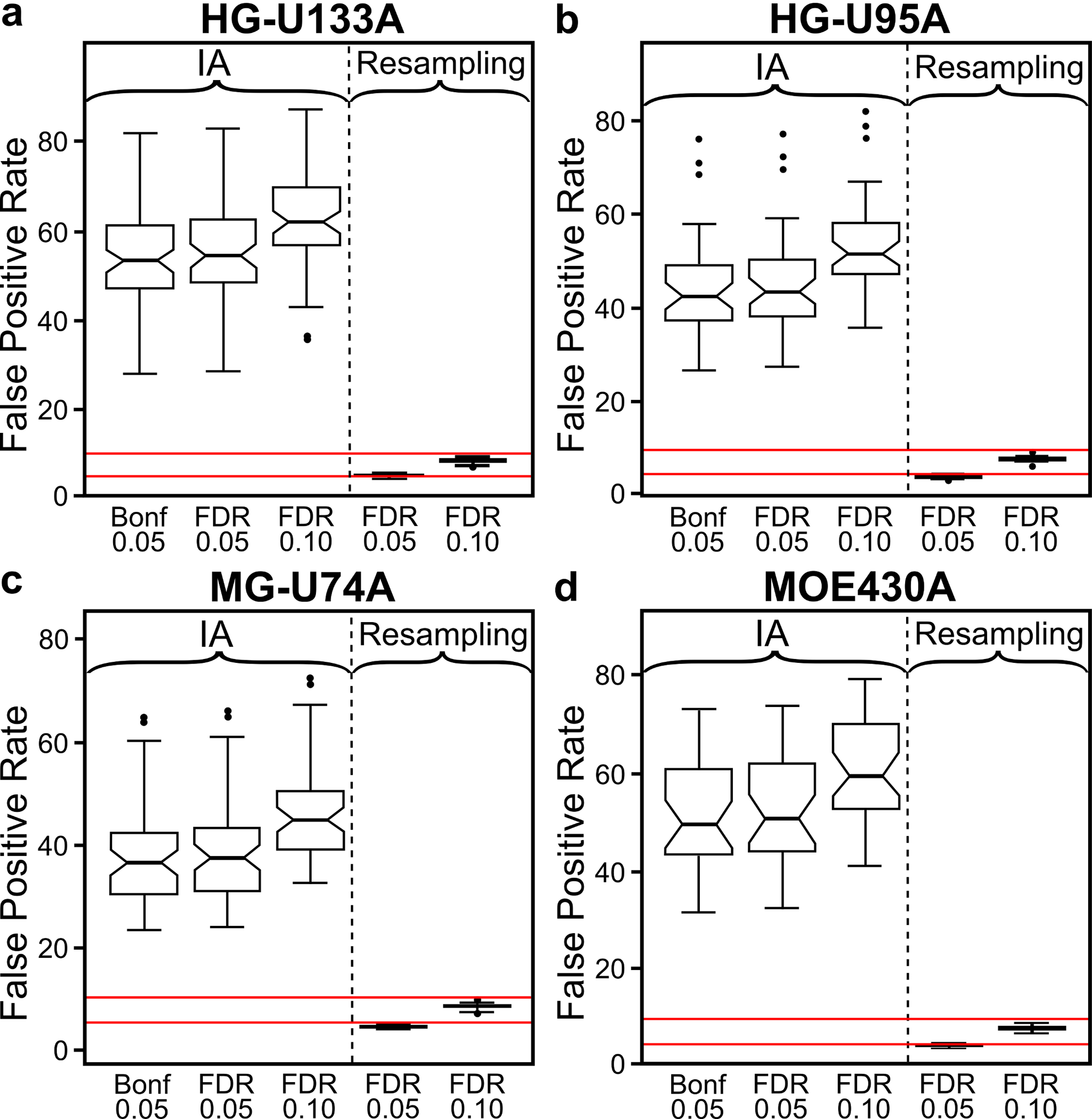
**

**Additional file 1, Figure S1.** **False positive rates are greatly increased using independence assumption methods, KEGG pathways.** The proportion of permutations in which at least one KEGG pathway is called significant using an independence assumption method with a Bonferroni correction (α = 0.05), the Benjamini & Hochberg FDR (α = 0.05, 0.10), and the resampling approach described in this manuscript. Red lines = 5% & 10%.


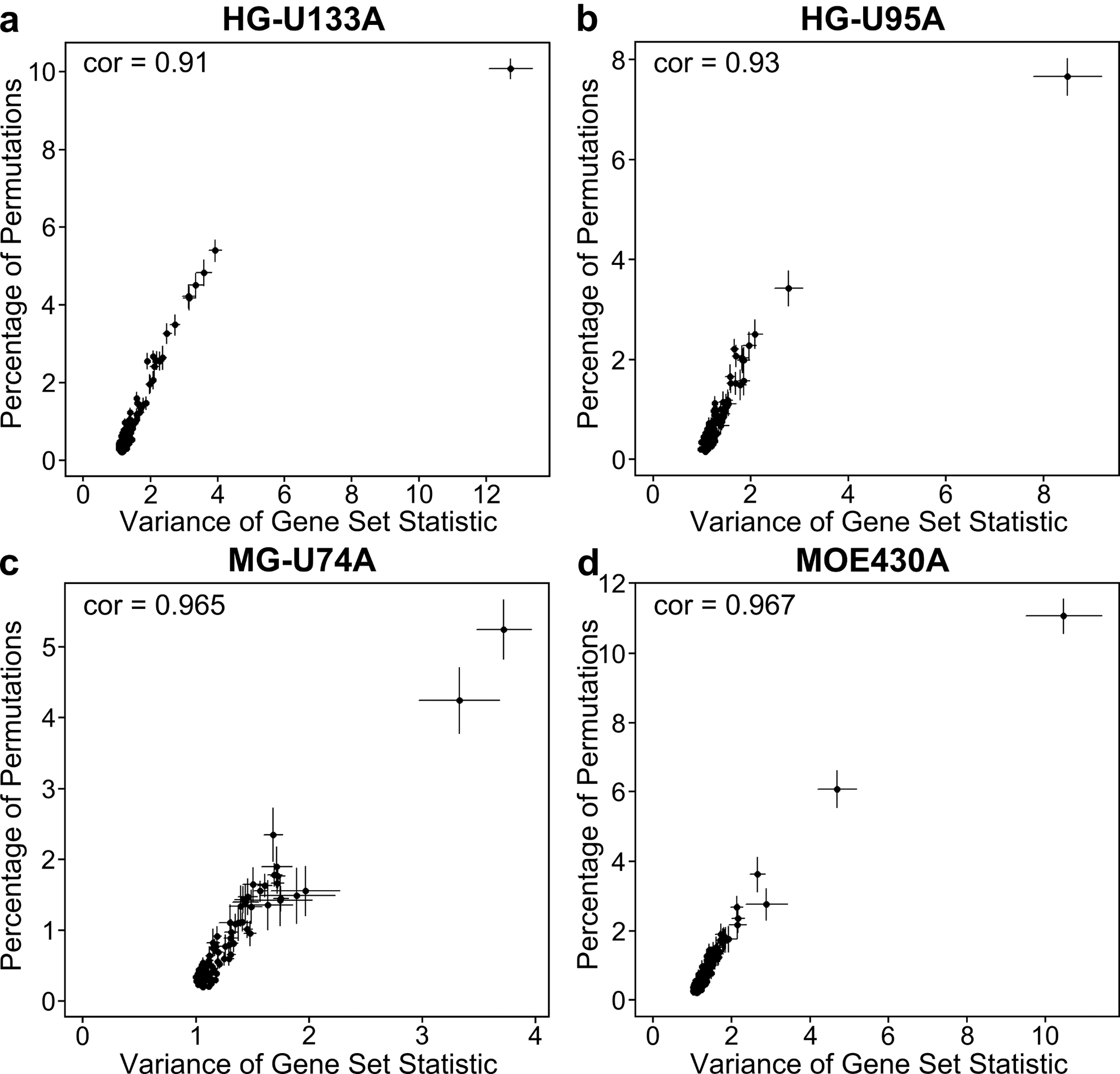


**Additional file 1, Figure S2. Variance inflation due to gene expression correlation increases the false positive rate, even when using a Bonferroni correction.** The percentage of permutations in which at least one KEGG pathway was called significant is plotted versus the variance of the standardized gene set statistic (signed square root of theχ^2^ statistic). Results are shown for two human (a,b) and two mouse (c,d) arrays. Spearman correlations are shown in the upper left of each panel.

**
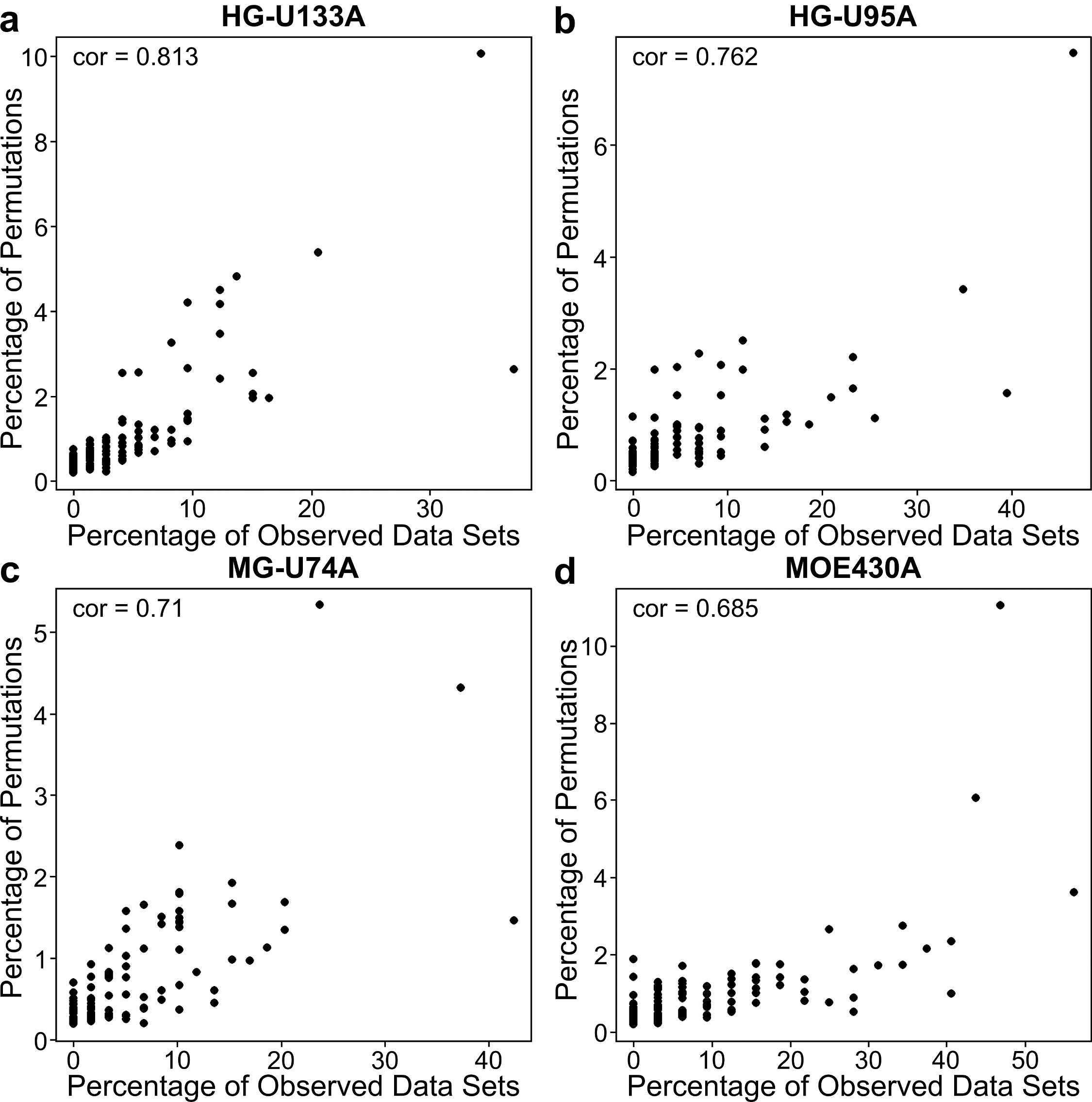
**

**Additional file 1, Figure S3.** **KEGG pathways that are called significant by chance under permutation are likely to be called significant in the observed data.** The proportion of times that a KEGG pathway is declared significant under permutation is plotted versus the proportion of times it is called significant in the observed data. Spearman correlations are shown in upper left corner.

**
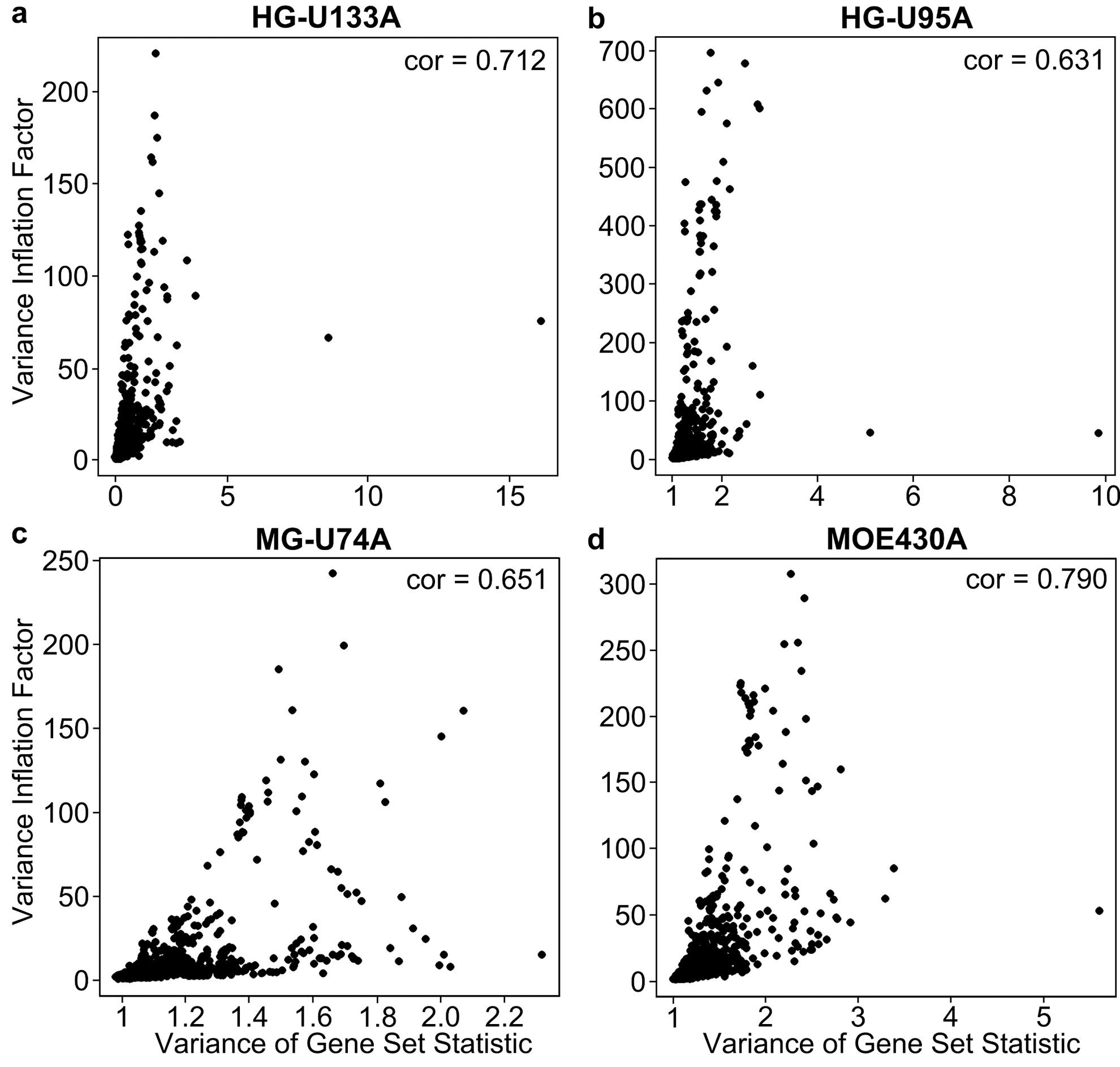
**

**Additional file 1, Figure S4.** **The variance of the gene set statistic (signed square root of χ^2^ statistic) increases in proportion to the variance inflation factor (VIF = 1 + (*m*-1)ρ).**  The VIF is plotted versus the variance of the gene set statistic versus for two human (a, b) and two mouse (c, d) arrays. Spearman correlations are shown in the upper right corner.
