# Supplementary material for: Heading Down the Wrong Pathway: on the Influence of Correlation within Gene Sets
Source: BMC Genomics. 2010 Oct 18;11:574. doi: 10.1186/1471-2164-11-574 (PMC3091509; doi:10.1186/1471-2164-11-574)
Supplement: Additional file 2 — Table S1. HG-U95A array datasets. PubMed, GEO and other IDs and descriptions of the datasets used. Additional File 2. Table S2. HG-U133A array datasets. PubMed, GEO and other IDs and descriptions of the datasets used. Additional File 2. Table S3. mgu74a array datasets. PubMed, GEO and other IDs and descriptions of the datasets used. Additional File 2. Table S4. moe430a array datasets. PubMed, GEO and other IDs and descriptions of the datasets used. [file 1471-2164-11-574-S2.DOC]

**Supplemental File 2. Table S1. HG-U95A array datasets.**

| **Array** | **GEO DS** | **Title** | **Description** | **PubMed ID** | **GPL** | **Feature Count** | **Organism** | **Sample Count** | **GSE** |
| --- | --- | --- | --- | --- | --- | --- | --- | --- | --- |
| HG-U95A | GDS181 | Large-scale analysis of the human transcriptome | Gene expression profiles from a diverse array of tissues, organs, and cell lines, from the normal physiological state. Represents a preliminary description of the normal mammalian transcriptome. | 11904358 | GPL91 | 12651 | Homo sapiens | 85 | GSE96 |
| HG-U95A | GDS360 | Breast cancer and docetaxel treatment | Breast cancer core biopsies taken from patients found to be resistant (greater than 25% residual tumor volume) or sensitive (less than 25% residual tumor volume) to docetaxel treatment. | NA | GPL91 | 12651 | Homo sapiens | 24 | GSE6434 |
| HG-U95A | GDS400 | DNA damage and UV radiation | Temporal analysis of differences in WS1 human skin fibroblast gene expression response to low (10 J/m2; induces transient cellular replicative arrest) or high (50 J/m2; induces apoptosis) doses of short wavelength UV radiation (UVC; 254 nm). | 12907719 | GPL91 | 12651 | Homo sapiens | 20 | GSE713 |
| HG-U95A | GDS422 | Normal human tissue expression profiling | Analysis of gene expression in a variety of normal tissues. Samples typically composed of a pool of 10-25 individuals. Included as part of the GeneNote project. | 15388519 | GPL91 | 12651 | Homo sapiens | 24 | GSE803 |
| HG-U95A | GDS449 | Cell cycle and Tat transactivation | Analysis of effect of Tat transactivation during cell cycle. HeLa CD4+ cells transfected with epitope-tagged Tat plasmid or parental vector pCEP4. Cells synchronized with cell cycle blocker hydroxyurea or nocodazole and sampled at 0, 3, 6 and 9 hours. | 15780141 | GPL91 | 12651 | Homo sapiens | 36 | GSE6431 |
| HG-U95A | GDS478 | Coxsackievirus B3 pathogenesis (II) | Temporal analysis of in vitro model of coxsackievirus B3 (CVB3) infection. HeLa cells infected with either CVB3, CVB3 and U0126 (blocks cytokines and metalloproteinases) or control PBS, and samples examined at 0, 0.5, 3 and 9 hours after treatment. | NA | GPL91 | 12651 | Homo sapiens | 24 | GSE697 |
| HG-U95A | GDS495 | Angiogenesis | Temporal analysis of human umbilical cord vein endothelial cell (HUVEC) isolates treated with angiogenic factors vascular endothelial growth factor-A (VEGF-A) and placental growth factor (PlGF) in low or high serum media. | NA | GPL91 | 12651 | Homo sapiens | 33 | GSE837 |
| HG-U95A | GDS531 | Multiple myeloma and bone lesions | Comparison of gene expression in bone marrow plasma cells of multiple myeloma patients with and without bone lesions. Osteolytic lesions increase in multiple myeloma patients. | 14695408 | GPL91 | 12651 | Homo sapiens | 173 | GSE755 |
| HG-U95A | GDS532 | Hydrostatic pressure-responsive genes in optic nerve head astrocytes | Temporal analysis of how astrocytes in the optic nerve head (ONH) respond to changes in intraocular pressure (IOP). ONH astrocytes exposed to 60 mm Hg hydrostatic pressure for 6, 24, and 48 hours. Lends understanding to pathogenesis of glaucoma. | 14747662 | GPL91 | 12651 | Homo sapiens | 44 | GSE758 |
| HG-U95A | GDS563 | Duchenne muscular dystrophy (II) (HG-U95A) | Search for modifying factors and pathogenic pathways involved in Duchenne muscular dystrophy (DMD). Quadricep skeletal muscle biopsies from 12 DMD patients and 12 unaffected control patients examined. | 12415109 | GPL91 | 12651 | Homo sapiens | 24 | GSE1004 |
| HG-U95A | GDS707 | Aging brain: frontal cortex expression profiles at various ages | Analysis of gene expression profiles obtained from the postmortem frontal cortex of 18 normal males and 12 normal females at 26 to 106 years of age. Results provide insight into molecular events underlying the onset of brain aging. | 15190254 | GPL91 | 12651 | Homo sapiens | 30 | GSE1572 |
| HG-U95A | GDS711 | Juvenile rheumatoid arthritis expression profiles in mononuclear cells | Examination of peripheral blood and synovial fluid mononuclear cells in pauciarticular and polyarticular rheumatoid arthritis, and in juvenile spondyloarthropathy. Results identify disease subtypes and provide insight into disease mechanisms. | 15150433 | GPL91 | 12651 | Homo sapiens | 57 | GSE1402 |
| HG-U95A | GDS724 | Kidney transplant rejection expression profiling | Analysis of kidney transplant rejection by expression profiling of kidney biopsies and peripheral blood lymphocytes from patients. Results identify expression profiles unique to rejection, dysfunction without rejection, and well-functioning transplants. | 15307835 | GPL91 | 12651 | Homo sapiens | 62 | GSE1563 |
| HG-U95A | GDS884 | Estradiol effect on osteosarcoma cells expressing estrogen receptor alpha or beta: time course | Expression profiling of U2OS osteosarcoma cells after treatment with 17beta-estradiol (E2) for various lengths of time up to 48 hours. U2OS transfected with either estrogen receptor (ER) alpha or beta. Results identify signaling pathways in bone regulat | 15033914 | GPL91 | 12651 | Homo sapiens | 20 | GSE1153 |
| HG-U95A | GDS913 | DNA damage from ultraviolet and ionizing radiation effect on peripheral blood lymphocytes | Analysis of peripheral blood lymphocytes exposed to ultraviolet (10 J/m^2) or ionizing radiation (5 Gy). Lymphocytes obtained from 15 healthy individuals from ages 21 to 36 years and immortalized with Epstein-Barr virus. Results provide insight into trans | 15356296 | GPL91 | 12651 | Homo sapiens | 45 | GSE1977 |
| HG-U95A | GDS963 | Macular degeneration and dermal fibroblast response to sublethal oxidative stress | Expression profiling of dermal fibroblasts from patients with macular degeneration. Fibroblasts subjected to sublethal oxidative stress. This study tests the hypothesis that patients with macular degeneration have an abnormal response to cellular injury | NA | GPL91 | 12651 | Homo sapiens | 36 | GSE1719 |
| HG-U95A | GDS968 | Radiation therapy toxicity association with abnormal transcriptional response to DNA damage | Analysis of UV and IR irradiated lymphoblastoid cell lines derived from the peripheral blood of patients with acute radiation toxicity. Samples taken 2 months after the completion of radiation therapy. Results provide insight into the role of the respon | 15096622 | GPL91 | 12651 | Homo sapiens | 171 | GSE1725 |
| HG-U95A | GDS971 | Rhabdomyosarcoma and Ewing's sarcoma comparison | Comparison of gene expression in 12 pediatric rhabdomyosarcomas (RMS) to that in 11 pediatric Ewing's sarcomas (EWS). 3 embryonic and 9 alveolar RMS tumors examined. RMS and EWS appear similar in routine histology. Results identify expression profiles | NA | GPL91 | 12651 | Homo sapiens | 23 | GSE967 |
| HG-U95A | GDS1020 | Post-traumatic stress disorder development | Expression profiling of peripheral mononuclear blood cells (PMBC) from patients with post-traumatic stress disorder. PMBC samples obtained from patients a few hours and 4 months after exposure to psychological stress. | 15685253 | GPL91 | 12651 | Homo sapiens | 33 | GSE860 |
| HG-U95A | GDS1021 | Renal cancer response to rapamycin analog CCI-779 treatment: time course | Expression profiling of peripheral blood mononuclear cells (PBMC) from patients with advanced renal cancer following treatment with the rapamycin analog CCI-779. Gene expression examined 8 and 16 weeks after treatment. Results identify potential gene mark | 15637533 | GPL91 | 12651 | Homo sapiens | 103 | GSE1982 |
| HG-U95A | GDS1059 | Acute myeloid leukemia response to chemotherapy | Analysis of mononuclear cells from 54 chemotherapy treated patients less than 15 years of age with acute myeloid leukemia (AML). Mononuclear cells taken from peripheral blood or bone marrow. Results identify expression patterns associated with complete re | 12738660 | GPL91 | 12651 | Homo sapiens | 58 | GSE2191 |
| HG-U95A | GDS1070 | Hypopharyngeal cancer at various stages of progression | Analysis of 34 hypopharyngeal cancer samples at various stages of progression. Samples taken from patients undergoing surgery. Hypopharyngeal cancers tend to be aggressive, and almost all are mucosal cell carcinomas. Results identify genes potentially inv | 14676830 | GPL91 | 12651 | Homo sapiens | 38 | GSE2379 |
| HG-U95A | GDS1212 | Tumor necrosis factor effect in the absence of NF-kappaB activity: time | Temporal analysis of the effect of tumor necrosis factor (TNF) on gene expression in HeLa cells with NF-kappaB inactivated. NF-kappaB inactivated with 2 ug/ml doxycycline. Cells examined at various time points following stimulation with 25 ng/ml TNF. Res | 15722553 | GPL91 | 12651 | Homo sapiens | 32 | GSE2624 |
| HG-U95A | GDS1221 | Chronic myelogenous leukemia response to imatinib | Analysis of peripheral blood and bone marrow of chronic myelogenous leukemia (CML) patients prior to imatinib (Gleevec) treatment. Imatinib induces complete cytogenetic response (CCR) in most CML patients. This study attempts to determine transcriptional | 15820940 | GPL91 | 12651 | Homo sapiens | 28 | GSE2535 |
| HG-U95A | GDS1256 | IFN-gamma inflammatory effect on bronchial epithelial cells modulated by dexamethasone: time course | Analysis of bronchial epithelial cells treated with interferon (IFN) gamma, or dexamethasone (dex), or both. IFN-gamma plays a role in lung inflammatory responses and corticosteroids such as dex are used as treatments for inflammation. Cells examined 8 or | 15985639 | GPL91 | 12651 | Homo sapiens | 36 | GSE1815 |
| HG-U95A | GDS1290 | CD4+ lymphocyte polarization into Th1 and Th2 cells in the presence of TGFbeta: time course | Analysis of CD4+ lymphocyte induced to differentiate into Th1 and Th2 by treatment with IL-12 and IL-4 respectively in the presence of TGFbeta. Cells examined at various time points up to 48 hours after treatment. Results provide insight into the mechanis | 14607935 | GPL91 | 12651 | Homo sapiens | 34 | GSE2770 |
| HG-U95A | GDS1388 | B-cell chronic lymphocytic leukemia progression | Analysis of primary lymphocytes from B-cell chronic lymphocytic leukemia (B-CLL) patients. Lymphocytes from patients with indolent B-CLL compared to those with progressive B-CLL. Results identify putative gene markers of B-CLL progression from a stable to | 16038780 | GPL91 | 12651 | Homo sapiens | 21 | GSE2403 |
| HG-U95A | GDS1442 | PPARÎ± agonist ciprofibrate effect on liver | Analysis of primate livers exposed to ciprofibrate at various doses for 4 or 15 days. Peroxisome proliferator-activated receptor-Î± (PPARÎ±) agonists such as ciprofibrate cause hepatocarcinogenesis in rodents but not in primates. Results identify species | 16081524 | GPL91 | 12651 | Macaca fascicularis | 23 | GSE2853 |
| HG-U95A | GDS1454 | B-cell chronic lymphocytic leukemia subtypes | Analysis of 100 genetically characterized B-cell chronic lymphocytic (B-CLL) leukemia samples. Genomic aberrations and variable heavy chain somatic mutations are important prognostic factors of B-CLL. Results identify expression signatures that define B-C | 15459216 | GPL91 | 12651 | Homo sapiens | 111 | GSE2466 |
| HG-U95A | GDS1493 | Obesity: adipocyte expression profile | Analysis of cultured abdominal subcutaneous mature adipocytes from 9 male and 10 female non-diabetic obese Pima Indians. The prevalence of obesity in Pima Indians is among the highest of any population. Results provide insight into the role of adipocytes | 16059715 | GPL91 | 12651 | Homo sapiens | 39 | GSE2508 |
| HG-U95A | GDS1650 | Pulmonary adenocarcinoma | Analysis of pulmonary adenocarcinomas (AC). Carcinogen exposure is responsible for the majority of ACs. Results compared with those obtained from a urethane-induced lung tumor model in the mouse (GDS1649), and provide insight into the conserved pathways u | 16314486 | GPL91 | 12651 | Homo sapiens | 39 | GSE2514 |
| HG-U95A | GDS1726 | HIV encephalitis: brain frontal cortex | Analysis of brain frontal cortex of HIV-seropositive patients with HIV encephalitis (HIVE). HIVE affects >40% of AIDS patients, promoting neurodegeneration and cognitive impairment. Results suggest HIV-mediated dysregulation of genes involved in neuronal | 15579294 | GPL91 | 12651 | Homo sapiens | 28 | GSE3489 |
| HG-U95A | GDS1887 | Rectal cancer cells and radiotherapy response | Analysis of rectal cancer cells from patients prior to preoperative radiotherapy. Response to radiotherapy determined by histopathologic examination of resected specimens. Results identify gene markers for the characterization and prediction of the respon | 16585155 | GPL91 | 12651 | Homo sapiens | 46 | GSE3493 |
| HG-U95A | GDS1954 | Various T7 RNA polymerase-based RNA amplification/labeling methods | Comparison of expression profiles generated using various T7 RNA polymerase-based in vitro transcription (IVT) RNA labeling methods. The length of the IVT reactions ranged from 4 to 16 hours. Results provide evidence for amplification method-dependent bia | 16645204 | GPL91 | 12651 | Homo sapiens | 20 | GSE3254 |
| HG-U95A | GDS2048 | Acute rotavirus infection: peripheral blood mononuclear cells | Expression profiling of peripheral blood mononuclear cells (PBMCs) from children with acute rotavirus diarrhea. Rotavirus is the most common cause of severe diarrhea in children. | 17267507 | GPL91 | 12651 | Homo sapiens | 23 | GSE2729 |
| HG-U95A | GDS2435 | Male and female venous blood | Comparison of venous blood of males to that of females. Results provide insight into the feasibility of using gene expression profiles to differentiate between genders and as a diagnostic tool. | NA | GPL91 | 12651 | Homo sapiens | 23 | GSE3115 |
| HG-U95A | GDS2520 | Head and neck squamous cell carcinoma | Analysis of paired normal tissues and tumor samples from patients with head and neck squamous cell carcinoma (HNSCC). Results used to assess the effectiveness of using a combinatorial approach to analyze microarray data in identifying differentially expr | 15170515 | GPL91 | 12651 | Homo sapiens | 44 | GSE6631 |
| HG-U95A | GDS2545 | Metastatic prostate cancer | Analysis of metastatic prostate tumors and primary prostate tumors. Normal tissue adjacent to the tumor and normal donor tissue also examined. Metastasis reflects the most adverse clinical outcome. Results provide insight into the molecular mechanisms un | NA | GPL91 | 12651 | Homo sapiens | 171 | GSE6919 |
| HG-U95A | GDS2642 | Crohnâ€™s disease and ulcerative colitis | Analysis of colonoscopic biopsies from patients with Crohnâ€™s disease (CD) or ulcerative colitis (UC). CD and UC are inflammatory bowel diseases with variable, overlapping clinical features and complex pathophysiologies. Results provide insight into the | 17262812 | GPL91 | 12651 | Homo sapiens | 36 | GSE6731 |
| HG-U95A | GDS2678 | Brain regions of humans and chimpanzees | Comparison of various human and chimpanzee brain regions. Results provide insight into the genetic basis of human specializations in brain organization and cognition. | 14557539 | GPL91 | 12651 | Pan troglodytes,Homo sapiens | 37 | GSE7540 |
| HG-U95A | GDS2785 | Ovarian cancer tumors after neo-adjuvant chemotherapy | Analysis of malignant ovarian cancer tumors from patients receiving neo-adjuvant chemotherapy. Ovarian adenomas and untreated ovarian carcinomas also examined. Results used to define gene expression signatures associated with clinical responses to chemot | 17505532 | GPL91 | 12651 | Homo sapiens | 43 | GSE7463 |
| HG-U95A | GDS2842 | Testicular seminoma progression | Analysis of testicular seminoma tumors at various stages of progression (pT1, pT2, pT3). Results provide insight into the pathogenesis of testicular seminoma. | 16158187 | GPL91 | 12651 | Homo sapiens | 43 | GSE8607 |
| HG-U95A | GDS2942 | Skeletal muscle disuse atrophy | Analysis of immobilized gastrocnemius muscles from patients with a leg placed in a cast to stabilize a fractured ankle. Results provide insight into the molecular mechanisms underlying muscle remodeling and atrophy due to muscle disuse. | 17804603 | GPL91 | 12651 | Homo sapiens | 28 | GSE8872 |

**Supplemental File 2. Table S2. HG-U133**A array datasets.

| Array Type | **GEO DS** | **Title** | **Description** | **PubMed ID** | **GPL** | **Feature Count** | **Organism** | **Sample Count** | **GSE** |
| --- | --- | --- | --- | --- | --- | --- | --- | --- | --- |
| HG-U133A | GDS266 | Asthma and atopy (HG-U133A) | Investigation of CD4+ lymphocytes from patients with and without atopy, in combination with asthma. | NA | GPL96 | 22283 | Homo sapiens | 29 | GSE473 |
| HG-U133A | GDS268 | Obesity and fatty acid oxidation | Identification of proteins involved in fatty acid oxidation in skeletal muscle. Fat oxidation may be reduced in morbidly obese individuals. Provides understanding of how obesity contributes to cardiovascular disease via insulin resistance. | 16849634 | GPL96 | 22283 | Homo sapiens | 24 | GSE474 |
| HG-U133A | GDS395 | Biomaterial engineering | Temporal gene expression analysis of fetal lung fibroblast cell line IMR-90 seeded onto collagen/chondroitin sulfate tissue engineering scaffold mesh material. Integration of genomic information with biomaterial engineering. | 15159079 | GPL96 | 22283 | Homo sapiens | 32 | GSE6432 |
| HG-U133A | GDS534 | Smoking-induced changes in airway transcriptome | Analysis of cigarette smoking-induced changes in bronchial epithelia, and reversibility of effects when smoking is discontinued. May provide insight to molecular events leading to chronic obstructive pulmonary disease (COPD) and lung cancer. | 15210990 | GPL96 | 22283 | Homo sapiens | 75 | GSE994 |
| HG-U133A | GDS564 | Sex specific transcription in hypothalamus | Examination of sex specific transcription in hypothalamus using postmortem samples from individuals averaging 70 years of age. | NA | GPL96 | 22283 | Homo sapiens | 23 | GSE1147 |
| HG-U133A | GDS596 | Large-scale analysis of the human transcriptome (HG-U133A) | Gene atlas of human protein-encoding transcriptome. Examined gene expression profiles from 79 physiologically normal tissues obtained from various sources. | 15075390 | GPL96 | 22283 | Homo sapiens | 158 | GSE1133 |
| HG-U133A | GDS686 | Store-operated calcium entry in HEK-293 cells | Comparison of HEK-293-derived monoclonal cell lines with high or low levels of store-operated Ca2+ entry (SOCE). Levels of SOCE confirmed by monitoring thapsigargin-stimulated Ba2+ entry. | 15623568 | GPL96 | 22283 | Homo sapiens | 22 | GSE1309 |
| HG-U133A | GDS690 | Intestinal epithelial cell immune response | Analysis of intestinal epithelial cell immune response. 14 day old colon adenocarcinoma cell lines Caco-2 and T84 stimulated 4 hours with flagellin, or lymphotoxin beta or TNF alpha cytokines. | NA | GPL96 | 22283 | Homo sapiens | 24 | GSE1474 |
| HG-U133A | GDS737 | Lung tissue from smokers with severe emphysema | Comparison of lung tissue from smokers with severe emphysema (removed at lung volume reduction surgery) and smokers with mild or no emphysema. Study provides insights into the pathogenesis of chronic obstructive pulmonary disease (COPD). | 15374838 | GPL96 | 22283 | Homo sapiens | 30 | GSE1650 |
| HG-U133A | GDS810 | Alzheimer's disease at various stages of severity | Expression profiling of brain hippocampi from 22 postmortem subjects with Alzheimer's disease (AD) at various stages of severity. 7, 8, and 7 subjects diagnosed with incipient, moderate, and severe AD respectively. Results provide insight into mechanism | 14769913 | GPL96 | 22283 | Homo sapiens | 31 | GSE1297 |
| HG-U133A | GDS946 | Familial combined hyperlipidemia | Expression profiling of lymphoblastic cell lines derived from the peripheral blood lymphocytes of patients with familial combined hyperlipidemia (FCHL), the most common form of genetically determined dyslipidemia. Results provide insight into the pathoph | NA | GPL96 | 22283 | Homo sapiens | 24 | GSE1010 |
| HG-U133A | GDS987 | Kidney transplant response to calcineurin inhibitor-free immunosuppression using sirolimus | Analysis of kidneys from adult renal transplant recipients subjected to calcineurin inhibitor-free immunosuppression using sirolimus. Patients treated with sirolimus have a lower prevalence of chronic allograft nephropathy compared to those treated with c | 15476476 | GPL96 | 22283 | Homo sapiens | 41 | GSE1743 |
| HG-U133A | GDS999 | Bronchoalveolar lavage cells of lung transplant recipients with acute rejection | Expression profiling of bronchoalveolar lavage cells of lung transplant recipients with acute rejection. Rejection status determined from biopsies. Results identify possible gene markers of lung transplant rejection. | 12958056 | GPL96 | 22283 | Homo sapiens | 34 | GSE2018 |
| HG-U133A | GDS1023 | Hematopoietic stem cell engraftment in goat | Human-goat chimerism achieved by transplanting human CD34+Lin- cord blood cells into fetal goats. Markers indicate human genes expressed in goat liver and blood. Human/goat hematopoietic stem cell (HSC) xenogeneic model allows analysis of HSC transplantat | 16682618 | GPL96 | 22283 | Homo sapiens,Capra hircus | 23 | GSE1801 |
| HG-U133A | GDS1036 | Microglial cell response to interferon-gamma: time course | Expression profiling of microglial cells obtained from 4 different samples following treatment with 200 u/ml interferon-gamma (IFN-gamma). Cells examined 1, 6, and 4 hours after treatment. Results provide insight into the regulation of immune functions | 16163375 | GPL96 | 22283 | Homo sapiens | 24 | GSE1432 |
| HG-U133A | GDS1062 | Squamous cell carcinoma of the oral cavity with lymph node metastasis | Expression profiling of 18 primary squamous cell carcinomas of the oral cavity with or without lymph node metastasis. Expression in lymph node metastasis also examined. Results used to identify gene expression signature in primary tumors that indicates th | 15558013 | GPL96 | 22283 | Homo sapiens | 27 | GSE2280 |
| HG-U133A | GDS1063 | Primary effusion lymphomas and associated viral infections | Analysis of Kaposi's sarcoma-associated herpesvirus (KSHV) positive mononuclear cells from 3 patients with primary effusion lymphoma (PEL), 9 KSHV positive PEL cell lines, and 3 KSHV negative cell lines from lymphomatous effusions. Effect of concomittant | NA | GPL96 | 22283 | Homo sapiens | 21 | GSE2149 |
| HG-U133A | GDS1064 | Acute myeloid leukemia subclasses | Expression profiling of bone marrow from 43 patients with various forms of acute myeloid leukemia (AML): 10 promyelocytic leukemias with t(15;17), 4 AMLs with inv(16), 7 monocytic leukemias, and 22 nonmonocytic leukemias. Results identify expression sign | 15674361 | GPL96 | 22283 | Homo sapiens | 43 | GSE1729 |
| HG-U133A | GDS1067 | Plasma cell dyscrasias | Expression profiling of plasma cells from patients with plasma cell dyscrasias: 7 with monoclonal gammopathy of undetermined significance, 39 with multiple myeloma (MM), and 6 with plasma cell leukemia. Results provide insight into the neoplastic transfo | 15735737 | GPL96 | 22283 | Homo sapiens | 52 | GSE2113 |
| HG-U133A | GDS1096 | Normal tissues of various types | Expression profiling of 36 types of normal tissue. Each RNA tissue sample pooled from several donors. Results identify tissue specific genes and provide baselines for interpreting gene expression in cancer. | 15950434 | GPL96 | 22283 | Homo sapiens | 36 | GSE2361 |
| HG-U133A | GDS1209 | Sarcoma and hypoxia | Expression profiling of soft tissue sarcoma samples. Hypoxic regions often develop in tumors as they increase in size. Results provide insight into the expression of hypoxia-related genes in sarcomas. |  | GPL96 | 22283 | Homo sapiens | 54 | GSE2719 |
| HG-U133A | GDS1220 | Malignant pleural mesothelioma | Expression profiling of malignant pleural mesothelioma (MPM) tumors. MPM is a highly lethal malignancy associated with asbestos exposure. Results provide insight into the pathogenesis of MPM. | NA | GPL96 | 22283 | Homo sapiens | 54 | GSE2549 |
| HG-U133A | GDS1257 | Sickle cell plasma effect on pulmonary artery endothelial cells (HG-U133A) | Expression profiling of pulmonary artery endothelial cells exposed to plasma from sickle cell disease (SCD) patients with sickle acute chest syndrome or from SCD patients at steady state. Results provide insight into the role of extra-erythrocytic factors | 15741505 | GPL96 | 22283 | Homo sapiens | 33 | GSE1849 |
| HG-U133A | GDS1282 | Clear cell sarcoma of the kidney | Expression profiling of clear cell sarcoma of the kidney (CCSK). Wilms' tumors and fetal kidney control samples also examined. Objective is to reveal diagnostic markers and insight into the pathogenesis of CCSK. | 16299227 | GPL96 | 22283 | Homo sapiens | 35 | GSE2712 |
| HG-U133A | GDS1284 | Multiple myeloma molecular classification | Analysis of CD138+ plasma cells purified from bone marrow of multiple myeloma (MM) patients. Results used to classify MM cases into translocation/cyclin (TC) groups based on cyclin D expression and presence of translocations in the immunoglobulin heavy ch | 16129847 | GPL96 | 22283 | Homo sapiens | 50 | GSE2912 |
| HG-U133A | GDS1321 | Barrett's metaplasia progression to adenocarcinoma | Expression profiling of normal esophageal epithelium, premalignant Barrett's metaplasia, and esophageal adenocarcinoma samples. Tissue samples of each type obtained from 8 patients by transhiatal esophagectomy. Results identify potential markers of diseas | 15833844 | GPL96 | 22283 | Homo sapiens | 24 | GSE1420 |
| HG-U133A | GDS1324 | T-cell acute lymphoblastic leukemia with CALM-AF10 fusion | Analysis of T-cell acute lymphoblastic leukemia (ALL) samples containing t(10;11)(p13;q14-21) translocation that fuses the clathrin-assembly protein-like lymphoid-myeloid leukemia gene CALM to the transcription factor AF10. Results provide insight into th | 16107895 | GPL96 | 22283 | Homo sapiens | 23 | GSE2117 |
| HG-U133A | GDS1329 | Molecular apocrine breast tumors | Analysis of tumors of 49 breast cancer patients. Tumors classified into luminal and basal classes, and a novel molecular apocrine class. Apocrine tumors are estrogen receptor negative (ER-) and androgen receptor positive (AR+), while luminal tumors are ER | 15897907 | GPL96 | 22283 | Homo sapiens | 49 | GSE1561 |
| HG-U133A | GDS1331 | Huntington's disease: peripheral blood expression profile (HG-U133A) | Analysis of blood samples of 5 presymptomatic and 12 symptomatic Huntington's disease (HD) patients. Studies suggest that gene expression may be altered in a variety of tissues in HD, including peripheral blood. Results identify potential markers for HD. | 16043692 | GPL96 | 22283 | Homo sapiens | 31 | GSE1751 |
| HG-U133A | GDS1362 | Ischemic and nonischemic cardiomyopathy comparison | Analysis of myocardial tissues from nonischemic (NICM) and ischemic cardiomyopathy (ICM) patients. NICM and ICM, major forms of dilated cardiomyopathy leading to congestive heart failure, have similar presentations but differ in pathophysiology, prognosis | 15769906 | GPL96 | 22283 | Homo sapiens | 37 | GSE1869 |
| HG-U133A | GDS1375 | Cutaneous malignant melanoma | Expression profiling of primary malignant melanoma, benign skin nevi, and normal skin samples. Results identify potential molecular markers for lymph node staging assays, and provide insight into melanoma tumorigenesis. | 16243793 | GPL96 | 22283 | Homo sapiens | 70 | GSE3189 |
| HG-U133A | GDS1390 | Prostate cancer progression after androgen ablation | Analysis of prostate cancer progression following androgen ablation treatment. 10 treated androgen-independent primary prostate tumors compared to 10 untreated androgen-dependent primary prostate tumors. Results provide insight into progression of prosta | 16203770 | GPL96 | 22283 | Homo sapiens | 20 | GSE2443 |
| HG-U133A | GDS1392 | Myelodysplastic syndrome | Analysis of bone marrow CD34+ progenitor cells from normal-karyotype, low-blast-count, early, low-risk myelodysplastic syndrome (MDS) patients, age-matched controls, and patients with non-MDS anemia. Results provide insight into pathogenesis of MDS and id | 16076868 | GPL96 | 22283 | Homo sapiens | 28 | GSE2779 |
| HG-U133A | GDS1476 | Uterine fibroids with fumarate hydratase mutations | Analysis of 7 uterine fibroids carrying fumarate hydratase (FH) mutations. FH catalyzes hydration of fumarate in the Krebs tricarboxylic acid cycle. Results provide insight into the connection between the disruption of mitochondrial metabolic pathways and | 16319128 | GPL96 | 22283 | Homo sapiens | 22 | GSE2152 |
| HG-U133A | GDS1479 | Carcinoma in situ lesions of the urinary bladder | Analysis of bladder biopsies of superficial transitional cell carcinomas with or without surrounding carcinoma in situ (CIS) lesions and muscle invasive carcinomas (mTCC). CIS is a common mTCC precursor. Results provide insight into which tumors in early | 15173019 | GPL96 | 22283 | Homo sapiens | 60 | GSE3167 |
| HG-U133A | GDS1480 | Obesity: preadipocyte expression profile (HG-U133A) | Analysis of cultured abdominal subcutaneous preadipocytes from 7 male and 7 female non-diabetic obese Pima Indians. The prevalence of obesity in Pima Indians is among the highest of any population. Results provide insight into the role of preadipocytes in | 16034612 | GPL96 | 22283 | Homo sapiens | 28 | GSE2510 |
| HG-U133A | GDS1584 | Oral squamous cell carcinoma | Analysis of oral squamous cell carcinoma (OSCC) cells from 16 patients. OSCC cells isolated from tumors by laser capture microdissection. Results identify a strong correlation between gene expression profile and tumor invasiveness in OSCC. | 15381369 | GPL96 | 22283 | Homo sapiens | 20 | GSE3524 |
| HG-U133A | GDS1615 | Ulcerative colitis and Crohn's disease comparison: peripheral blood mononuclear cells | Analysis of peripheral blood mononuclear cells (PBMCs) from Crohn's disease (CD) and ulcerative colitis (UC) patients. Results identify a PBMC expression signature that distinguishes between CD and UC, suggesting that diagnosis of these two inflammatory b | 16436634 | GPL96 | 22283 | Homo sapiens | 127 | GSE3365 |
| HG-U133A | GDS1618 | Lymph node and tonsil comparison | Analysis of lymph node (sinuses) and tonsil (no sinuses), highly similar secondary lymphoid organs. Metastatic tumor cells are preferentially arrested in the lymph node sinuses. Results identify signature genes that are prime candidates for mediating adhe | 16440291 | GPL96 | 22283 | Homo sapiens | 20 | GSE2665 |
| HG-U133A | GDS1663 | Expression data from different research centers | Comparison of expression data from the Translational Genomics Research Institute to that from the Children's National Medical Center in Washington, DC. Data consists of normal kidney, liver, and spleen expression profiles. | NA | GPL96 | 22283 | Homo sapiens | 25 | GSE2004 |
| HG-U133A | GDS1688 | Various lung cancer cell lines | Expression profiling of a set of 29 lung cancer cell lines consisting of 10 non-small cell adenocarcinoma, 10 small cell cancer, and 9 squamous cell cancer lines. Gene expression results analyzed in relation to the sensitivity of each cell line to common | NA | GPL96 | 22283 | Homo sapiens | 29 | GSE4127 |
| HG-U133A | GDS1746 | Primary epithelial cell cultures from prostate tumors | Analysis of epithelial cell cultures from prostate tumor explants. Results identify an epithelial-restricted transcription profile that can be integrated with tumor grade and clinical information with the aim of discriminating indolent and aggressive pros | 16513839 | GPL96 | 22283 | Homo sapiens | 30 | GSE3868 |
| HG-U133A | GDS1815 | High-grade gliomas (HG-U133A) | Analysis of high-grade glioma (HGGs) samples from cases of WHO grade III and IV astrocytomas. Results classify HGGs into molecular subclasses with prognostic value that predict survival and disease progression. The molecular subclasses resemble key stages | 16530701 | GPL96 | 22283 | Homo sapiens | 100 | GSE4271 |
| HG-U133A | GDS1902 | Cyanobacterial metabolite apratoxin A cytotoxic effect on colon adenocarcinoma cells: time course and dose response | Analysis of colon adenocarcinoma HT29 cells at various time points up to 12 hours following treatment with 2 or 10 nM of the cyanobacterial metabolite apratoxin A. Results provide insight into the molecular mechanisms underlying the cytotoxic effect of ap | 16474387 | GPL96 | 22283 | Homo sapiens | 27 | GSE2742 |
| HG-U133A | GDS1926 | Leukotriene D4 and thrombin effect on endothelial cells: time course | Temporal analysis of umbilical vein endothelial cells (HUVEC) treated with leukotriene D4 (LTD4) and/or thrombin. LTD4 activates the cysteinyl leukotriene receptor cysLT2-R. Thrombin activates the protease-activated receptor PAR-1. cysLT2-R and PAR-1 may | 16606835 | GPL96 | 22283 | Homo sapiens | 21 | GSE3589 |
| HG-U133A | GDS1956 | Various muscle diseases (HG-U133A) | Analysis of muscle biopsy specimens from patients with various muscle diseases. Results provide insight into the diagnosis and pathogenesis of muscle diseases. | 16478798 | GPL96 | 22283 | Homo sapiens | 121 | GSE3307 |
| HG-U133A | GDS1972 | RNAlater preservative effect on uterine myometrium: time course | Analysis of uterine myometrial tissue specimens stored in RNAlater preservative for 24 or 74 hours at room temperature. Results indicate RNALater did not contribute any systematic shift in quantitative RNA expression results relative to alternative tissue | 15537428 | GPL96 | 22283 | Homo sapiens | 24 | GSE1296 |
| HG-U133A | GDS1975 | Gliomas of grades III and IV (HG-U133A) | Analysis of grades III and IV gliomas of various histologic types. Results used to develop a gene-expression based, histology independent-classification scheme, and provide insight into the biology of gliomas. | 15374961 | GPL96 | 22283 | Homo sapiens | 85 | GSE4412 |
| HG-U133A | GDS2055 | Skeletal muscle types (HG-U133A) | Analysis of tibialis anterior, deltoid, quadriceps, and gastrocnemius muscles obtained at autopsy from pediatric and geriatric subjects unaffected by neuromuscular disease. Results provide insight into the molecular basis of the selective involvement of d | 15962335 | GPL96 | 22283 | Homo sapiens | 35 | GSE4667 |
| HG-U133A | GDS2113 | Pheochromocytomas of various genetic origins | Analysis of 76 adrenal and extra-adrenal pheochromocytomas. These neural crest-derived tumors of uniform phenotype arise from inherited or sporadic mutations in at least six independent genes. Results provide insight into the molecular pathogenesis of phe | 16103922 | GPL96 | 22283 | Homo sapiens | 75 | GSE2841 |
| HG-U133A | GDS2190 | Bipolar disorder: dorsolateral prefrontal cortex | Analysis of postmortem dorsolateral prefrontal cortex from 30 adults with bipolar disorder. Results provide insight into the pathophysiology of the disease. | 16894394 | GPL96 | 22283 | Homo sapiens | 61 | GSE5388 |
| HG-U133A | GDS2191 | Bipolar disorder: orbitofrontal cortex | Analysis of postmortem orbitofrontal cortex from 10 adults with bipolar disorder. Results provide insight into the pathophysiology of the disease. | 16894394 | GPL96 | 22283 | Homo sapiens | 21 | GSE5389 |
| HG-U133A | GDS2201 | Serrated and conventional adenocarcinomas | Comparison of serrated and conventional colorectal adenocarcinoma tumor samples. Results differentiate serrated from conventional colorectal carcinomas (CRC) and provide insight into the molecular pathogenesis of serrated CRC. | 16819509 | GPL96 | 22283 | Homo sapiens | 37 | GSE4045 |
| HG-U133A | GDS2214 | Septic neutrophil response to lipopolysaccharide and high mobility group box 1 protein | Analysis of septic neutrophils treated with lipopolysaccharide or high mobility group box 1 (HMGB1) protein. Neutrophils isolated from patients with sepsis-induced acute lung injury (ALI). HMGB1 is a late mediator of endotoxin lethality, and neutrophils p | NA | GPL96 | 22283 | Homo sapiens | 24 | GSE3037 |
| HG-U133A | GDS2255 | Transmigrated neutrophils in the alveolar space of endotoxin-exposed lung | Analysis of neutrophils that transmigrate to the alveolar space. Transmigration induced by bronchoscopic instillation of volunteers with endotoxin (LPS). Results identify differences between pulmonary and circulating neutrophils that occur early in endoto | 16861384 | GPL96 | 22283 | Homo sapiens | 58 | GSE2322 |
| HG-U133A | GDS2297 | Gefitinib effect on various non-small cell lung cancer cell lines (HG-U133A) | Analysis of baseline non-small cell lung cancer (NSCLC) cell lines with a broad range of sensitivity to the anticancer drug gefitinib. Gefitinib is an epidermal growth factor receptor (EGFR) tyrosine kinase inhibitor (TKI). Results used to define a gene e | 16877703 | GPL96 | 22283 | Homo sapiens | 45 | GSE4342 |
| HG-U133A | GDS2324 | Low concentrations of 17beta-estradiol effect on breast cancer cell line | Analysis of breast cancer MCF7/BUS cells treated with 17beta-estradiol (E2) at concentrations up to 100 pM. Results compared to that from an experiment examining the response to hormone starvation (GDS2323). Results provide insight into the E2 sensitivity | 14610279 | GPL96 | 22283 | Homo sapiens | 25 | GSE4668 |
| HG-U133A | GDS2341 | Type I and Type II interferons effect on lung epithelial cell line: time course | Analysis of lung epithelial A549 cells 6 and 24 hours following treatment with type I interferon (IFN), type II IFN, or both. Results provide insight into the molecular basis of the synergistic antiviral and antiproliferative effects observed when type I | 16800785 | GPL96 | 22283 | Homo sapiens | 32 | GSE5542 |
| HG-U133A | GDS2362 | Presymptomatic and symptomatic malaria: peripheral blood mononuclear cells | Comparison of peripheral blood mononuclear cells of subjects with early, presymptomatic, experimentally acquired malaria to those with acute, uncomplicated, naturally acquired malaria. Results provide insight into the immune response to malaria in these t | 16988231 | GPL96 | 22283 | Homo sapiens | 71 | GSE5418 |
| HG-U133A | GDS2366 | Preadipocytes from anatomically separate fat depots (HG-U133A) | Analysis of undifferentiated and differentiated preadipocytes from abdominal subcutaneous, mesenteric, and greater omental regions. Separate fat depots differ in size, function, and contribution to pathological states. Results provide insight into the con | 16985259 | GPL96 | 22283 | Homo sapiens | 24 | GSE1657 |
| HG-U133A | GDS2373 | Squamous cell lung carcinomas | Analysis of primary squamous cell lung carcinomas (SCCs) from 129 patients. SCCs and adenocarcinomas compose the majority of non small cell lung cancers. Gene expression profiles were compared to clinical outcome. | 16885343 | GPL96 | 22283 | Homo sapiens | 130 | GSE4573 |
| HG-U133A | GDS2493 | Glucocorticoid sensitive and resistant acute lymphoblastic leukemia samples | Analysis of pretreatment acute lymphoblastic leukemia samples that are either sensitive or resistant to glucocorticoid (GC)-induced apoptosis in vitro. Results used to search a database of expression profiles of pharmacologic treatment of cells for small | 17010674 | GPL96 | 22283 | Homo sapiens | 29 | GSE5820 |
| HG-U133A | GDS2516 | Interferons effect on endothelial cells | Analysis of endothelial cells treated with interferon (IFN) alpha, beta, or gamma for 5 hours. The effect of IFNs on fibroblasts was also examined. Results provide insight into the molecular basis of the antiangiogenic activity exhibited by IFNs. | 17202376 | GPL96 | 22283 | Homo sapiens | 23 | GSE3920 |
| HG-U133A | GDS2518 | Psoriatic plaques | Analysis of uninvolved and lesional skin of 13 patients with plaque-type psoriasis. Psoriasis vulgaris is characterized by hyperproliferation and incomplete terminal differentiation of epidermal keratinocytes. Results provide insight into the molecular pa | 16858420 | GPL96 | 22283 | Homo sapiens | 26 | GSE6710 |
| HG-U133A | GDS2519 | Early-stage ParkinsonΓÇÖs disease: whole blood | Analysis of blood of 50 patients predominantly with early-stage ParkinsonΓÇÖs disease (PD). Results provide insight into the molecular processes perturbed in the cellular blood of patients with early-stage PD. | 17215369 | GPL96 | 22283 | Homo sapiens | 105 | GSE6613 |
| HG-U133A | GDS2528 | Basal plate of the placenta from midgestation to term (HG-U133A) | Analysis of the basal plate of the placenta from midgestation to term. The basal plate is the region where maternal and fetal cells coexist. Its proper formation is required for pregnancy. Results provide insight into the molecular basis of the unique cel | 17170095 | GPL96 | 22283 | Homo sapiens | 36 | GSE5999 |
| HG-U133A | GDS2643 | Waldenstrom's macroglobulinemia: B lymphocytes and plasma cells | Analysis of B lymphocytes (BL) and plasma cells (PC) from patients with Waldenstrom's macroglobulinemia (WM), a B-lymphoproliferative disorder (BLPD). Results provide insight into differences between PC and BL from WM and their cell counterpart in chronic | 17252022 | GPL96 | 22283 | Homo sapiens | 56 | GSE6691 |
| HG-U133A | GDS2649 | HIV infection effect on CD4+ and CD8+ T cells | Analysis of CD4+ and CD8+ T cells from HIV patients at different clinical stages and rates of disease progression. Changes in T-cell function are a hallmark of HIV infection. Results provide insight into the pathogenic mechanisms in HIV infections that le | 17251300 | GPL96 | 22283 | Homo sapiens | 40 | GSE6740 |
| HG-U133A | GDS2655 | Fetal and adult reticulocytes (HG-U133A) | Analysis of circulating blood reticulocytes from umbilical cords and normal adults. Reticulocytes are immature red blood cells containing residual RNA. Results provide insight into the molecular basis of terminal erythroid differentiation. | 17405831 | GPL96 | 22283 | Homo sapiens | 28 | GSE6236 |
| HG-U133A | GDS2736 | Malignant fibrous histiocytoma and various soft tissue sarcomas | Analysis of malignant fibrous histiocytoma (MFH) tumors and various soft tissue sarcomas (STSs). MFH is characterized by non-recurrent complex chromosomal aberrations, and overlapping histological and immunohistochemical phenotypes. Results provide insigh | 17464315 | GPL96 | 22283 | Homo sapiens | 105 | GSE6481 |
| HG-U133A | GDS2767 | Blood response to various beverages: time course | Analysis of blood samples from 6 individuals at various time points up to 12 hours following the intake of water, ethanol, grape juice, or red wine. Results may contribute to elucidating the mechanisms underlying the cardioprotective effects of red wine. | 17010189 | GPL96 | 22283 | Homo sapiens | 108 | GSE3846 |
| HG-U133A | GDS2771 | Large airway epithelial cells from cigarette smokers with suspect lung cancer | Analysis of large airway epithelial cells from cigarette smokers without cancer, with cancer, and with suspect lung cancer. Results provide insight into the feasibility of using gene expression to detect early stage lung cancer in smokers. | 17334370 | GPL96 | 22283 | Homo sapiens | 192 | GSE4115 |
| HG-U133A | GDS2777 | Bexarotene effect on gemcitabine resistant non-small lung cancer cell line | Analysis of gemcitabine (Gem)-resistant non-small lung cancer Calu3 cells treated with bexarotene (Bex). Acquired drug resistance is a major obstacle in cancer therapy. Results provide insight into molecular mechanisms underlying the ability of bexarotene | 17483357 | GPL96 | 22283 | Homo sapiens | 20 | GSE6914 |
| HG-U133A | GDS2922 | Ascending aortic aneurysms | Analysis of aneurysmal tissue from ascending aortas of patients with normal tricuspid aortic valves or abnormal bicuspid aortic valves (BAVs). BAV is a congenital anomaly associated with an increased risk for ascending aortic aneurysm (AscAAs). Results pr | 17502243 | GPL96 | 22283 | Homo sapiens | 25 | GSE5180 |

**Supplemental File 2. Table S3. mgu74a** array datasets.

| Array Type | **GEO DS** | **Title** | **Description** | **PubMed ID** | **GPL** | **Feature Count** | **Organism** | **Sample Count** | **GSE** |
| --- | --- | --- | --- | --- | --- | --- | --- | --- | --- |
| mgu74a | GDS144 | Cardiac hypertrophy, exercise-induced | Effects of chronic conditioning by swimming which increases left ventricular mass and induces myocyte hypertrophy. 8 week old FVB mice swum in tanks 7 days a week for 4 weeks. Animals sacrificed at several time points up to 28 days. | NA | GPL81 | 12488 | Mus musculus | 30 | GSE77 |
| mgu74a | GDS200 | Contextual fear conditioning paradigm | Mice subject to novel spatial context with 0.5 mA shock, shock alone or control. Alzheimer's mice (50% C57BL/6, 50% SJL) and MK-801-injected mice also examined. Dentate gyrus and CA1 hippocampus tissue examined 1, 2, 4 or 6 hours after treatment. | 15102909 | GPL81 | 12488 | Mus musculus | 64 | GSE1782 |
| mgu74a | GDS234 | Muscle regeneration (U74Av2) | Cardiotoxin injected into gastrocnemius muscle to induce muscle regeneration. Muscles profiled at 27 time points (0-40 days) post-injection. Transcriptional regulatory pathways defined. | 12023284 | GPL81 | 12488 | Mus musculus | 54 | GSE469 |
| mgu74a | GDS241 | Alveoli destruction time course | Temporal study of lung alveoli destruction induced by caloric restriction. Alveoli number and surface area are linked to oxygen consumption, and calorie restriction lowers oxygen consumption. | 14594731 | GPL81 | 12488 | Mus musculus | 24 | GSE478 |
| mgu74a | GDS251 | Pulmonary fibrosis | Determination of genetic basis of sensitivity to pulmonary fibrosis induced by bleomycin by comparing susceptible (C57BL6/J) and resistant (Balb/c) strains. C57BL6/J experience inflammatory response then progressive lung disease, Balb/c do not. | NA | GPL81 | 12488 | Mus musculus | 24 | GSE485 |
| mgu74a | GDS488 | Myocardial infarction time course | Temporal analysis of acute myocardial infarction (AMI) induced by left coronary artery ligation. Left ventricle above and below left anterior descending (LAD) artery examined at 1 hour to 8 week time points. | NA | GPL81 | 12488 | Mus musculus | 59 | GSE775 |
| mgu74a | GDS586 | Myogenic differentiation timecourse (MG-U74A) | Analysis of early stages of myogenesis. C2C12 myoblasts induced to differentiate and examined during cell proliferation (days -2 to -1), at cell cycle withdrawal (day 0) and during myogenic fusion and maturation of multinucleated myotubes (days 2 to 10). | 14688207 | GPL81 | 12488 | Mus musculus | 24 | GSE989 |
| mgu74a | GDS591 | Tetracycline transactivator expression in heart | Determination of effects of tetracycline transactivator (tTA) expression in cardiac ventricle of FVBN mice. | 15797971 | GPL81 | 12488 | Mus musculus | 20 | GSE986 |
| mgu74a | GDS604 | Neurofibromatosis and neurodevelopment | Examination of hippocampus from 10 to 32 day old NF1 heterozygotes. NF1+/- mice develop learning and memory difficulties mimicking cognitive deficits in human neurofibromatosis. | NA | GPL81 | 12488 | Mus musculus | 30 | GSE1482 |
| mgu74a | GDS605 | Spermatogenesis and testis development time course (MG-U74A) | Spermatogenesis time course generated from BL/6-129 testis collected from birth through adulthood. Provides insight into genes implicated in maturation, maintenance, and function of testis and the integrated process of spermatogenesis. | 15028632 | GPL81 | 12488 | Mus musculus | 22 | GSE926 |
| mgu74a | GDS614 | Dystrophin-deficient mdx extraocular muscle development time course | Analysis of extraocular muscle (EOM) from dystrophin-deficient mdx mice, a Duchenne muscular dystrophy (DMD) model. Postnatal ages 14, 28, 56, and 112 days examined. EOM is unaffected in DMD, so results provide insight into mdx EOM protective mechanisms. | 12874102 | GPL81 | 12488 | Mus musculus | 24 | GSE1008 |
| mgu74a | GDS638 | Dystrophin-deficient mdx diaphram muscle development time course | Temporal analysis of diaphram muscle from dystrophin-deficient mdx mice, a Duchenne muscular dystrophy (DMD) model. Postnatal ages 7 to 112 days examined. Results provide insight into mechanisms of muscular dystrophy pathogenesis. | 14681298 | GPL81 | 12488 | Mus musculus | 36 | GSE1026 |
| mgu74a | GDS639 | Dystrophin-deficient mdx hindlimb muscle development time course | Temporal analysis of hindlimb gastrocnemius/soleus muscle from dystrophin-deficient mdx mice, a Duchenne muscular dystrophy (DMD) model. Postnatal ages 7 to 112 days examined. Results provide insight into mechanisms of muscular dystrophy pathogenesis. | 12874102 | GPL81 | 12488 | Mus musculus | 36 | GSE1025 |
| mgu74a | GDS640 | Germ-free gastrointestinal tract | Analysis of germ-free and conventional corpus, jejunum, descending colon and rectum. Findings identify important interaction between commensal microbiota and gastrointestinal tract transcriptome. | 15226484 | GPL81 | 12488 | Mus musculus | 24 | GSE1392 |
| mgu74a | GDS641 | Dystrophin-deficient mdx, mdx5cv and wild type skeletal muscle profiles | Analysis of diaphragm, extensor digitorum longus, gastroc, soleus, quadricep, and tibialis anterior muscle in 8 week old male dystrophin-deficient mdx, mdx5cv, and C57BL10 wild type provides insight into pathogenesis of muscular dystrophy. | NA | GPL81 | 12488 | Mus musculus | 36 | GSE897 |
| mgu74a | GDS773 | Retinoic acid teratogenic effect on cranial neural crest: time course | Expression profiling of cranial neural crest exposed to 1 uM retinoic acid (RA) at various lengths of time up to 48 hours. Neural crest obtained from 8.5 days postcoitum ICR embryos. Results provide insight into developmental pathways affected upon expo | 15466718 | GPL81 | 12488 | Mus musculus | 27 | GSE1588 |
| mgu74a | GDS794 | Cardiac hypertrophy progression: time course | Expression profiling of hearts from FVB males subjected to cardiac pressure overload by transverse aortic constriction (TAC). TAC performed on 3 month old males. Hearts examined 2, 10, and 21 days after surgery. Results provide insight into the progress | 15292486 | GPL81 | 12488 | Mus musculus | 26 | GSE1621 |
| mgu74a | GDS828 | Insulitis: comparison of innocuous and destructive forms | Analysis of CD4+ automimmune T and CD45+ hematopoeitic cells from non-obese diabetic (NOD) and C57BL/6-H-2 g7 transgenics with the BDC2.5 T cell receptor (TCR) from a diabetogenic T cell. BDC2.5 TCR in NOD and in C57BL/6-H-2 g7 results in innocuous and d | 15141080 | GPL81 | 12488 | Mus musculus | 24 | GSE1085 |
| mgu74a | GDS879 | X-linked hypophosphatemia and low phosphate diet | Examination of kidney from normal or X-linked hypophosphatemic (Hyp) 5 week old mice fed either control (1.0% P) or low phosphate diet (0.03% P) for 3 or 5 days. Results provide insight into mechanism of renal adaptation to low phosphate diet. | 15054142 | GPL81 | 12488 | Mus musculus | 20 | GSE868 |
| mgu74a | GDS882 | Neuromedin U effect on type-2 Th cells: time course | Analysis of type-2 Th cell line D10.G4.1 after treatment with 10 nM neuromedin U (NmU) for various times up to 12 hours. NmU is a neuropeptide that binds to its receptor on D10.G4.1 cells, eliciting the release of cytokines. This effect suggests a role fo | 15585845 | GPL81 | 12488 | Mus musculus | 31 | GSE1791 |
| mgu74a | GDS912 | Hair follicle development: time course | Expression profiling of back skin excised from CB6F1 animals at various ages up to 1 year. Difference between synchronously and asynchronously growing hair used to identify hair cycle genes. Results provide insight into the mechanisms regulating hair fo | 15520371 | GPL81 | 12488 | Mus musculus | 25 | GSE1912 |
| mgu74a | GDS981 | Uterine response to physiologic and plant-derived estrogen: time course | Temporal analysis of immature Alpk:APfCD-1 uterus response up to 72 hours after a single dose of physiologic estrogen 17beta-estradiol (E2) or phytoestrogen genistein (GEN). | 15289156 | GPL81 | 12488 | Mus musculus | 21 | GSE1819 |
| mgu74a | GDS997 | Transcription factor CHOP null mutation effect on fibroblasts subjected to ER stress: time course | Expression profiling of embryonic fibroblasts deleted for the transcription factor CHOP. Fibroblast treated with 2 ug/ml tunicamycin for 4 or 8 hours to induce endoplasmic reticulum (ER) stress. Results provide insight into molecular mechanisms regulatin | 15601821 | GPL81 | 12488 | Mus musculus | 24 | GSE2082 |
| mgu74a | GDS1010 | Bone marrow and muscle Side Population cell comparison | Expression profiling of Side Population (SP) cells from bone marrow and muscle of 4 to 5 week old C57BL/10SnJ males. Main Population from bone marrow and muscle also examined. Results provide insight into the similarities and differences between SP cell | NA | GPL81 | 12488 | Mus musculus | 20 | GSE1571 |
| mgu74a | GDS1058 | Uterus response to 17beta-estradiol: time course | Analysis of uteri of immature 19 to 20 day old Alpk:APfCD-1 females given a single injection of 400 ug/kg 17beta-estradiol (E2). Gene expression examined at various time points up to 72 hours following treatment. Results provide insight into mechanisms un | 15598610 | GPL81 | 12488 | Mus musculus | 42 | GSE2195 |
| mgu74a | GDS1077 | Hematopoietic stem cells from different recombinant inbred strains | Expression profiling of Lin- Sca-1+ c-kit+ hematopoietic stem cells (HSC) from 22 different BXD recombinant inbred (RI) strains. Each RI strain is homozygous for alleles at about 98% of loci. Results combined with QTL mapping to identify candidate genes f | 15711547 | GPL81 | 12488 | Mus musculus | 44 | GSE2031 |
| mgu74a | GDS1219 | Insulin receptor substrate inactivation effect on brown preadipocytes | Expression profiling of brown preadipocytes derived from insulin receptor substrate (IRS) knockout animals.The IRS family of insulin signaling mediators is composed of IRS-1, IRS-2, IRS-3, and IRS-4. Results provide insight into the regulation of brown ad | 15895078 | GPL81 | 12488 | Mus musculus | 28 | GSE2556 |
| mgu74a | GDS1247 | Dysferlin deficiency effect on skeletal and cardiac muscles | Comparison of skeletal and cardiac muscles of dysferlin deficient SJL/J animals. Dysferlin deficiency results in skeletal muscle weakness, but does not affect the heart. Dysferlin mutations can result in the neuromuscular disorders Limb-Girdle muscular dy | 16237120 | GPL81 | 12488 | Mus musculus | 20 | GSE2507 |
| mgu74a | GDS1261 | GH/IGF-1 signaling disruption and caloric restriction additive effect | Analysis of livers from growth hormone/insulin-like growth factor-1 signaling defective Ames dwarfs (DF) subjected to caloric restriction (CR). DF and CR in combination additively extend life span. Results provide insight into the mechanisms underlying th | 15039484 | GPL81 | 12488 | Mus musculus | 31 | GSE1093 |
| mgu74a | GDS1298 | Fibroblast adipogenesis induced by EBF-1 and PPARgamma2 overexpression: time course | Analysis of NIH-3T3 embryonic fibroblasts induced to differentiate into adipocytes by overexpression of early B cell factor (EBF)-1 or peroxisome proliferator-activated receptor (PPAR)gamma2 transcription factors. Cells examined at various time points up | 16106032 | GPL81 | 12488 | Mus musculus | 53 | GSE2192 |
| mgu74a | GDS1302 | 2,3,7,8-tetrachlorodibenzo-p-dioxin effect on cardiovascular development | Analysis of gestational day (GD) 17.5 fetal hearts isolated from pregnant C57BL/6Ns treated at GD 14.5 with up to 6.0 ug/kg 2,3,7,8-tetrachlorodibenzo-p-dioxin (TCDD). TCDD, a cardiovascular teratogen, inhibits cardiomyocyte proliferation, a process that | 16120747 | GPL81 | 12488 | Mus musculus | 20 | GSE2812 |
| mgu74a | GDS1371 | Peripheral myelin protein 22 gene dosage and point mutation effect on sciatic nerve | Analysis of sciatic nerve from postnatal day 4 and 60 mutants overexpressing peripheral myelin protein 22 (PMP22), lacking PMP22, or expressing the Trembler PMP22 point mutation. Results provide insight into normal function of PMP22, myelination, and neur | 15755691 | GPL81 | 12488 | Mus musculus | 21 | GSE1947 |
| mgu74a | GDS1406 | Brain regions of various inbred strains | Analysis of 7 brain regions of 6 inbred strains. 7 week old males from 129S6/SvEvTac, A/J, C3H/HeJ, C57BL/6J, DBA/2J, and FVB/NJ strains examined. Results correlated with behavioral phenotypes and identify candidate genes for anxiety-like behavior. | 16244648 | GPL81 | 12488 | Mus musculus | 87 | GSE3327 |
| mgu74a | GDS1455 | Motoneuron characterization | Analysis of lateral and medial motoneurons (MNs) from lumbar segments and sympathetic preganglionic motoneurons located in the thoracic intermediolateral nucleus. MNs isolated by laser capture microdissection. Results provide insight into the molecular di | 16317082 | GPL81 | 12488 | Mus musculus | 30 | GSE2595 |
| mgu74a | GDS1490 | Neural tissue profiling | Expression profiling of 24 neural tissues and 10 body regions from adult A/J, C57BL/6J, C3H/HeJ, DBA/2J, and 129S6/SvEvTac males. Relationship of regional gene expression in the brain to architecture and organization of the brain examined. | 16002470 | GPL81 | 12488 | Mus musculus | 150 | GSE3594 |
| mgu74a | GDS1634 | Nodose and dorsal root ganglia comparison (MG-U74A) | Comparison of whole nodose ganglia (NG) and dorsal root ganglia (DRG) vs. the population of laser-captured visceral sensory neurons. Results identify striking differences and the need for microdissection when studying visceral sensory neurons because of d | 16303873 | GPL81 | 12488 | Mus musculus | 22 | GSE2917 |
| mgu74a | GDS1649 | Urethane-induced lung tumor model of pulmonary adenocarcinoma | Analysis of lung tumors that develop following treatment with urethane. Urethane-induced lung tumors exhibit similar histological appearance and molecular changes to human pulmonary adenocarcinoma (AC). Results compared with those from human AC (GDS1650). | 16314486 | GPL81 | 12488 | Mus musculus | 44 | GSE2514 |
| mgu74a | GDS1805 | Lactating mammary gland response to diets with varying amounts of fat: time course | Analysis of mammary glands from FVBs fed a diet with 8%, 16%, or 40% fat. Gene expression examined at pregnancy day 12 to lactation day 9. Results provide insight into whether multiple metabolic control points are involved in activating lipid and lactose | 17105756 | GPL81 | 12488 | Mus musculus | 32 | GSE4222 |
| mgu74a | GDS1808 | Candidate calorie restriction mimetic drugs effect on the liver | Analysis of livers of long-lived B6C3F1 animals fed a diet containing metformin, glipizide, rosiglitazone, or soy isoflavone extract. Results compared to hepatic gene expression profile produced by long-term caloric restriction. Caloric restriction extend | 16189280 | GPL81 | 12488 | Mus musculus | 32 | GSE2431 |
| mgu74a | GDS1845 | Retinitis pigmentosa 1 gene knockout effect on retinas: time course | Analysis of retinas of animals disrupted for the retinitis pigmentosa 1 (RP1) gene. Animals examined up to postnatal day 21. Results provide insight into mechanisms underlying autosomal dominant progressive RP caused by RP1 mutations and identify molecule | 16126734 | GPL81 | 12488 | Mus musculus | 30 | GSE128 |
| mgu74a | GDS1878 | Myotube response to PGC-1alpha induced mitochondrial biogenesis: time course (MG-U74A) | Analysis of C2C12 myoblast-derived myotubes transduced with transcriptional coactivator PGC-1alpha to induce mitochondrial proliferation in the muscle cells. Cells examined up to 3 days post-transduction. Results combined with other genome-scale datasets | 16582907 | GPL81 | 12488 | Mus musculus | 21 | GSE4330 |
| mgu74a | GDS1903 | Classical fear conditioning effect on amygdala and hippocampus | Comparison of amygdalae and hippocampi of animals subjected to classical fear conditioning (CFC), which consists of a 3-minute exposure to the conditioning chamber, a 30-second exposure to tone, and a 2-second, 1.5-mA footshock. CFC induced greater gene e | 16547164 | GPL81 | 12488 | Mus musculus | 48 | GSE3963 |
| mgu74a | GDS1957 | Interferon gamma effect on suppressor of cytokine signaling-1 deficient livers: time course | Analysis of the effect of interferon gamma (IFNg) on the livers of animals lacking IFNg, or both suppressor of cytokine signaling-1 (SOCS1) and IFNg. Gene expression examined up to 48 hours following IFNg treatment. Results provide insight into the role o | 16473883 | GPL81 | 12488 | Mus musculus | 22 | GSE4232 |
| mgu74a | GDS2025 | Myc activation and deactivation effect on pancreatic islet beta cells | Analysis of transgenic pancreatic islet beta cells after activation of Myc for up to 24 hours or activation for 21 days followed by deactivation for up to 6 days. Results provide insight into the molecular basis of Myc-driven tumorigenesis in vivo. | 16651409 | GPL81 | 12488 | Mus musculus | 27 | GSE4356 |
| mgu74a | GDS2082 | Age effect on the hippocampus | Analysis of hippocampi of 2-month-old young and 15-month-old middle-aged animals. The hippocampal formation is one of the brain areas most affected by aging. Results provide insight into molecular changes accompanying the early stages of age-related memor | 15169854 | GPL81 | 12488 | Mus musculus | 23 | GSE5078 |
| mgu74a | GDS2169 | Nuclear and extranuclear mutant huntingtin exon 1 protein effect on cerebellum | Analysis of cerebella of transgenics expressing mutant huntingtin (htt) exon 1 protein in nucleus only or in both nucleus and cytoplasm. The polyglutamine (polyQ) expansion in htt results in Huntington's disease (HD). Results provide insight into the impa | 16183657 | GPL81 | 12488 | Mus musculus | 48 | GSE3248 |
| mgu74a | GDS2177 | Type 1 diabetes model: autoimmune target organs (MG-U74A) | Analysis of pancreata and submandibular and lacrimal glands from 6-, 9-, and 15-week-old NOD/scid animals, respectively, before the onset of autoimmune destruction of the tissues. The NOD/scid animal is a model for type 1 diabetes. The three organs examin | 16895987 | GPL81 | 12488 | Mus musculus | 39 | GSE4953 |
| mgu74a | GDS2218 | Developing lung response to oxygen deficiency: time course | Analysis of developing lungs of animals subjected to hypoxia at 10% oxygen. Animals subjected to hypoxia at fetal day 21 or at birth for 2 or 6 hours. Results provide insight into the relationship between oxygen deficiency and gene expression in the perin | NA | GPL81 | 12488 | Mus musculus | 33 | GSE4310 |
| mgu74a | GDS2226 | Hippocampus of various inbred strains | Analysis of hippocampi from 8 inbred strains. The hippocampus is a key area of the brain involved in behaviors such as learning, memory, anxiety, and aggression. Results provide insight into strain-specific gene expression differences and their relationsh | 15128411 | GPL81 | 12488 | Mus musculus | 32 | GSE5429 |
| mgu74a | GDS2312 | Nemaline myopathy model: various skeletal muscles | Analysis of various skeletal muscles of trangenics expressing the human Met9Arg alpha-tropomyosin slow mutant gene, a model of nemaline myopathy (NM). NM, a non-dystrophic congenital myopathy, is a variably severe neuromuscular disorder. Results provide i | 16877500 | GPL81 | 12488 | Mus musculus | 36 | GSE3384 |
| mgu74a | GDS2329 | Acute myocardial infarction model: time course (MG-U74A) | Analysis of left heart ventricles (LV) at various time points up to 48 hours following surgically induced acute myocardial infarction (AMI). Ischemic/infarcted tissue, surviving free wall, and the interventricular septum of LV examined. Results provide in | 16845475 | GPL81 | 12488 | Mus musculus | 66 | GSE4648 |
| mgu74a | GDS2434 | Scavenger receptor A mutant olfactory epithelium response to olfactory bulbectomy: time course | Analysis of olfactory epithelia (OE) of scavenger receptor A (SR-A) mutants at up to 48 hours after olfactory bulbectomy (OBX). OBX induces apoptotic death of olfactory sensory neurons and recruitment of macrophages to the OE. SR-A mediates the binding of | 16882882 | GPL81 | 12488 | Mus musculus | 30 | GSE3455 |
| mgu74a | GDS2562 | Prostate response to castration and subsequent hormone replacement | Analysis of prostate of animals following castration and subsequent hormone replacement with testosterone. Castration induces prostate involution, while hormone replacement induces regeneration. Results provide insight into the molecular mechanisms regula | 17288544 | GPL81 | 12488 | Mus musculus | 20 | GSE5901 |
| mgu74a | GDS2654 | Neurological aging models: retinas and hippocampi | Analysis of retinas and hippocampi of SAMP8 and SAMP10 animals at 3 and 16 months of age. SAMP8 and SAMP10 are inbred animal models of accelerated neurological senescence. Results provide insight into the molecular mechanisms underlying neurological agin | 15960800 | GPL81 | 12488 | Mus musculus | 27 | GSE6238 |
| mgu74a | GDS2701 | Contraction- and freeze-injured skeletal muscles: time course | Analysis of skeletal muscles at various time points up to 7 days following injury caused by eccentric contraction (CI) or by freezing (FI). Results provide insight into the molecular events accompanying the degeneration and repair phases of contraction- a | 17478534 | GPL81 | 12488 | Mus musculus | 25 | GSE5413 |
| mgu74a | GDS2743 | Brown and white adipocyte differentiation | Comparison of brown and white preadipocytes at the undifferentiated and differentiating stages. Unlike white adipocytes, brown adipocytes have an abundance of mitochondria and are thus able to contribute to energy expenditure. Results provide insight into | 17360536 | GPL81 | 12488 | Mus musculus | 24 | GSE7032 |
| mgu74a | GDS2843 | Mammary gland development | Analysis of mammary glands during pregnancy, lactation, and involution. Results provide insight into the molecular mechanisms regulating mammary gland development. | 17338830 | GPL81 | 12488 | Mus musculus | 40 | GSE8191 |
| mgu74a | GDS2850 | Brain trauma model: time course (MG-U74A) | Analysis of brain at various time points up to 72 hours following lateral controlled cortical impact injury. Delayed cell death following trauma results in brain damage. Results provide insight into the mechanisms underlying brain damage after traumatic i | 14588109 | GPL81 | 12488 | Mus musculus | 22 | GSE2392 |
| mgu74a | GDS2936 | Neural retina leucine zipper deficiency effect on retinas: time course | Analysis of retinas of mutants lacking the rod photoreceptor-specific neural retina leucine zipper protein (Nrl), at up to 2 months of age. In the absence of Nrl, rods are missing and cones increase in number. Results provide insight into the differences | 15163632 | GPL81 | 12488 | Mus musculus | 24 | GSE8972 |

**Supplemental File 2. Table S4. moe430a array datasets.**

| Array Type | **GEO DS** | **Title** | **Description** | **PubMed ID** | **GPL** | **Feature Count** | **Organism** | **Sample Count** | **GSE** |
| --- | --- | --- | --- | --- | --- | --- | --- | --- | --- |
| moe430a | GDS565 | Sex specific transcription in somatic and reproductive tissues | Analysis of sex specific transcription in somatic and reproductive tissues. Hypothalamus, liver, kidney, ovary, and testis tissues pooled from 10 different animals examined. | NA | GPL339 | 22690 | Mus musculus | 48 | GSE1148 |
| moe430a | GDS813 | Preimplantation embryo development (MOE430A) | Expression profiling of CF-1 x B6D2F1/J preimplantation embryos. Oocytes and embryos at the 1-cell, 2-cell, 8-cell, and blastocyst stages examined. Results provide insight into mechanisms underlying the major transitions in preimplantation development. | 15282163 | GPL339 | 22690 | Mus musculus | 20 | GSE1749 |
| moe430a | GDS960 | Estrogen effect on lung: time course | Expression profiling of lungs from ovariectomized animals 3 and 6 hours following estrogen treatment. Estrogen prevents diminished alveoli formation in ovariectomized immature animals, and reverses the loss of alveoli in ovariectomized adults. | NA | GPL339 | 22690 | Mus musculus | 28 | GSE1303 |
| moe430a | GDS1030 | IL-21 and IL-15 synergistic effect on naÃ¯ve CD8+ T cells | Expression profiling of naÃ¯ve CD8+ T cells stimulated with IL-15 or IL-21 or both cytokines for 4 hours. 100 ng/ml of each cytokine used. Results provide insight into the synergistic effect of IL-21 and IL-15 on T cell homeostasis and effector function | NA | GPL339 | 22690 | Mus musculus | 20 | GSE2059 |
| moe430a | GDS1244 | Phosgene effect on lungs: time course | Analysis of lungs exposed to the toxic industrial compound carbonyl chloride (phosgene). Phosgene exposure results in pulmonary edema and acute lung injury. Lungs examined at several time points up to 72 hours after exposure. Provides insight into molecul | 16300373 | GPL339 | 22690 | Mus musculus | 104 | GSE2565 |
| moe430a | GDS1265 | Oocyte development (MOE430A) | Analysis of oocytes from follicles at the primordial to large antral stages of development, collected from B6SJLF1 animals at 2 to 22 days of age. Provides insight into the development of eggs of high meiotic and developmental competence. | 16168984 | GPL339 | 22690 | Mus musculus | 20 | GSE3351 |
| moe430a | GDS1276 | Inflammatory lung injury and mechanical ventilation | Analysis of lungs of C57BL/6 based model of lung inflammation and injury. The model develops acute lung injury (ALI) after concurrent exposure to low-dose lipopolysaccharide (LPS) and mechanical ventilation (MV). MV may contribute to ALI in critically ill | 16116230 | GPL339 | 22690 | Mus musculus | 24 | GSE2411 |
| moe430a | GDS1277 | Obliterative bronchiolitis and tracheal allograft | Analysis of tracheal grafts from BALB/c donors transplanted into MHC incompatible C57BL/6 recipients. Allografts examined up to 25 days after transplantation. Provides insight into pathogensis of allograft rejection and associated obliterative bronchiolit | 16095496 | GPL339 | 22690 | Mus musculus | 35 | GSE3418 |
| moe430a | GDS1309 | Myotube response to hydrogen peroxide | Expression profiling of myotubes following treatment with hydrogen peroxide at various concentrations up to 1000 uM. Myotubes derived from C2C12 myoblasts. Results provide insight into the response of muscle to reactive oxygen species. | NA | GPL339 | 22690 | Mus musculus | 20 | GSE3078 |
| moe430a | GDS1409 | cAMP/protein kinaseA effect on cell-cycle regulation: timecourse | Analysis of wild type and Kin- S49 lymphoma cell line treated with 8-(4-chlorophenylthio)-cAMP (8-CPT-cAMP), a PKA-selective cAMP analog, for 2, 6, or 24 hours. Kin- S49 cells lack protein kinase A (PKA). Results identify the global impact of cAMP/PKA on | 15939874 | GPL339 | 22690 | Mus musculus | 26 | GSE2413 |
| moe430a | GDS1465 | IFN-gamma and host genetic background effect on Yersinia enterocolitica infected macrophages | Analysis of bone marrow derived macrophages from resistant C57BL/6 or susceptible BALB/c animals, with or without IFN-gamma pretreatment, infected with Yersinia enterocolitica. Results provide insight into the impact of host genetic background and IFN-gam | 16352694 | GPL339 | 22690 | Mus musculus | 37 | GSE2973 |
| moe430a | GDS1492 | Bleomycin effect on lungs: dose response and time course | Comparison of lungs from C57BL/6J and C3H/HeJ strains, 3 and 6 weeks after treatment with 80, 100, or 125 units of bleomycin/kg. C57BL/6Js are susceptible to bleomycin-induced pulmonary fibrosis, while C3H/HeJs are resistant. Results identify candidate ge | 15937080 | GPL339 | 22690 | Mus musculus | 21 | GSE2640 |
| moe430a | GDS1522 | Forebrain neuronal subtypes | Analysis of 12 distinct neuronal subpopulations from 5 regions of the adult forebrain. Results identify highly heterogeneous expression profiles that likely underlie the fundamental differences observed between neuronal classes and between glutamatergic a | 16369481 | GPL339 | 22690 | Mus musculus | 42 | GSE2882 |
| moe430a | GDS1607 | Muscle glycogen content effect on skeletal muscle | Analysis of skeletal muscles of MGSKO and GSL30 lines which lack or over-accumulate muscle glycogen, respectively. Liver was also analyzed in MGSKO to assess whether muscle glycogen loss has indirect effects on liver. The two largest glycogen stores in ma | 16356168 | GPL339 | 22690 | Mus musculus | 34 | GSE2198 |
| moe430a | GDS1678 | Quercetin effect on hepatic cytochrome P-450 oxidoreductase null mutants | Analysis of cytochrome-P450 oxidoreductase (POR) null mutants at 24 hours following consumption of anti-carcinogenic flavonoid, quercetin. Results identify a diminished transcriptomic response in KO compared to WT in all tissues examined and provide insig | 16455785 | GPL339 | 22690 | Mus musculus | 48 | GSE4262 |
| moe430a | GDS1756 | CAG repeat knock-in model of spinocerebellar ataxia type 1 progression | Analysis of cerebellum and forebrain tissue of knock-in mice carrying 154 CAG repeats in the spinocerebellar ataxia type 1 (SCA1) locus at 4 and 12 weeks of age. Results provide insight into the pathophysiology of the neurodegenerative disease SCA1. | NA | GPL339 | 22690 | Mus musculus | 20 | GSE2867 |
| moe430a | GDS1765 | Extraocular and hindlimb skeletal muscle cell differentiation: time course (MG-430A) | Analysis of cell lines derived from extraocular (EOM) and gastrocnemius skeletal muscle allotypes at various time points up to 48 hours following induction of myogenesis. Results provide insight into the early developmental mechanisms underlying the forma | 16291736 | GPL339 | 22690 | Mus musculus | 30 | GSE4463 |
| moe430a | GDS1784 | Protein kinase B alpha knockout effect on adipogenesis: time course | Analysis of protein kinase B alpha (PKBalpha) knockout embryonic fibroblasts (MEFs) following treatment with a standard induction cocktail to induce differentiation into adipocytes. Cells examined up to 48 hours following treatment. Results provide insigh | 16478789 | GPL339 | 22690 | Mus musculus | 36 | GSE2746 |
| moe430a | GDS2135 | Prefrontal cortex postnatal development: time course | Analysis of prefrontal cortex (PFC) of animals at 2 to 10 weeks of age. Although the basic laminar structure of the PFC is established in utero, extensive remodeling continues into adolescence. Results map the overall pattern of changes in cortical gene t | 17013924 | GPL339 | 22690 | Mus musculus | 23 | GSE4675 |
| moe430a | GDS2139 | UV irradiation effect on skin inactivated for tyrosine kinase receptor ErbB2: time course | Analysis of the dorsal skin of animals treated with the tyrosine kinase receptor Erbb2 inhibitor AG825 and subsequently exposed to UV radiation. Skin examined 6 and 12 hours after UV irradiation. Results provide insight into the role of Erbb2 in the respo | 17003495 | GPL339 | 22690 | Mus musculus | 23 | GSE4066 |
| moe430a | GDS2158 | Emery-Dreifuss muscular dystrophy model: regenerating skeletal muscle | Analysis of regenerating gastrocnemius muscles of emerin and lamin A/C knockout mutants at various time points up to 4 days following treatment with cardiotoxin to induce muscle injury. Mutations in emerin and lamin A/C cause Emery-Dreifuss muscular dystr | NA | GPL339 | 22690 | Mus musculus | 23 | GSE5304 |
| moe430a | GDS2334 | Myod and Myog expression effect on myogenesis: time course | Temporal analysis of embryonic Myf-5/Myod null fibroblasts tranduced with a Myod-estrogen receptor hormone binding domain fusion protein alone or in combination with a constitutively expressed Myog. Results provide insight into the roles of Myod and Myog | 16437161 | GPL339 | 22690 | Mus musculus | 36 | GSE3858 |
| moe430a | GDS2464 | Polyglutamine expansion disease model: retina | Analysis of retinas from R7E animals expressing the mutant ataxin-7, which harbors 90 glutamines. Polyglutamine expansion in ataxin-7 causes spinocerebellar ataxia type 7 (SCA7). Results provide insight into the molecular and cellular pathways involved in | 17005371 | GPL339 | 22690 | Mus musculus | 30 | GSE3634 |
| moe430a | GDS2482 | Ras-associated binding protein 3A mutations effect on brain cortices and hippocampi (MG-430A) | Analysis of brain cortices and hippocampi of ras-associated binding protein (Rab) 3A D77G point or knockout mutants. Rab3a is a neuronal GTP-binding protein that binds synaptic vesicles and regulates synaptic transmission. The D77G point mutant exhibits a | 16734774 | GPL339 | 22690 | Mus musculus | 36 | GSE6527 |
| moe430a | GDS2524 | Effect of gonadal steroids on sex differences in response to blood-stage malaria infection: time course | Analysis of white blood cells (WBCs) isolated from spleens of intact and gonadectomized (gdx) males and females up to 14 days after inoculation with Plasmodium chabaudi. Results provide insight into molecular mechanisms mediating the sexual dimorphism in | 16714546 | GPL339 | 22690 | Mus musculus | 48 | GSE4324 |
| moe430a | GDS2531 | Antipsychotic agents clozapine and haloperidol effects on the brain (II) | Analysis of brains of animals treated for 12 weeks with the atypical antipsychotic clozapine or the typical antipsychotic haloperidol. Clozapine's use as an antipsychotic is limited by agranulocytosis. Results provide insight into the molecular basis of c | NA | GPL339 | 22690 | Mus musculus | 30 | GSE6467 |
| moe430a | GDS2554 | Diffuse large B cell lymphoma model | Analysis of lymphomas of transgenics engineered to constitutively express the dual bromodomain protein Brd2, which causes a malignancy similar to diffuse large B cell lymphoma (DLCL). Results provide insight into the similarity of DLCL lymphomas to activa | 17166848 | GPL339 | 22690 | Mus musculus | 26 | GSE6136 |
| moe430a | GDS2666 | Embryonic R1 stem cell differentiation in vitro (MG-430A) | Analysis of R1 embryonic stem cells differentiating into embryoid bodies in vitro. Cells examined at various time points up to 14 days after inducing differentiation. Results provide insight into the molecular mechanisms that drive embryonic stem cell dif | 17394647 | GPL339 | 22690 | Mus musculus | 33 | GSE2972 |
| moe430a | GDS2668 | Embryonic J1 stem cell differentiation in vitro (MG-430A) | Analysis of J1 embryonic stem cells differentiating into embryoid bodies in vitro. Cells examined at various time points up to 14 days after inducing differentiation. Results provide insight into the molecular mechanisms that drive embryonic stem cell dif | 17394647 | GPL339 | 22690 | Mus musculus | 33 | GSE3749 |
| moe430a | GDS2671 | Embryonic V6.5 stem cell differentiation in vitro (MG-430A) | Analysis of V6.5 embryonic stem cells differentiating into embryoid bodies in vitro. Cells examined at various time points up to 14 days after inducing differentiation. Results provide insight into the molecular mechanisms that drive embryonic stem cell d | 17394647 | GPL339 | 22690 | Mus musculus | 33 | GSE3231 |
| moe430a | GDS2945 | Islet amyloid polypeptide effect on pancreatic cell line: time course and dose response | Analysis of MIN6 pancreatic beta-cells treated for up to 24 hours with various concentrations of human islet amyloid polypeptide (IAPP). IAPP aggregration contributes to the development of islet amyloidosis in type 2 diabetes. IAPP oligomers are associate | 17563070 | GPL339 | 22690 | Mus musculus | 20 | GSE2253 |
| moe430a | GDS3202 | Comparative analysis of SCA1 and SCA7 knock-in models | Analysis of cerebellum in spinocerebellar ataxia type 1 (SCA1) and type 7 (SCA7) knock-in models. SCA1 and SCA7 patients manifest cerebellar ataxia with degeneration of Purkinje cells. Results provide insight into the shared molecular pathogenic response | 18216249 | GPL339 | 22690 | Mus musculus | 22 | GSE9914 |
